# Supplementary material for: The small CRL4CSA ubiquitin ligase component DDA1 regulates transcription-coupled repair dynamics
Source: Nat Commun. 2024 Jul 29;15:6374. doi: 10.1038/s41467-024-50584-7 (PMC11286758; doi:10.1038/s41467-024-50584-7)
Supplement: Supplementary file 1 — Supplementary Information [file 41467_2024_50584_MOESM1_ESM.pdf]

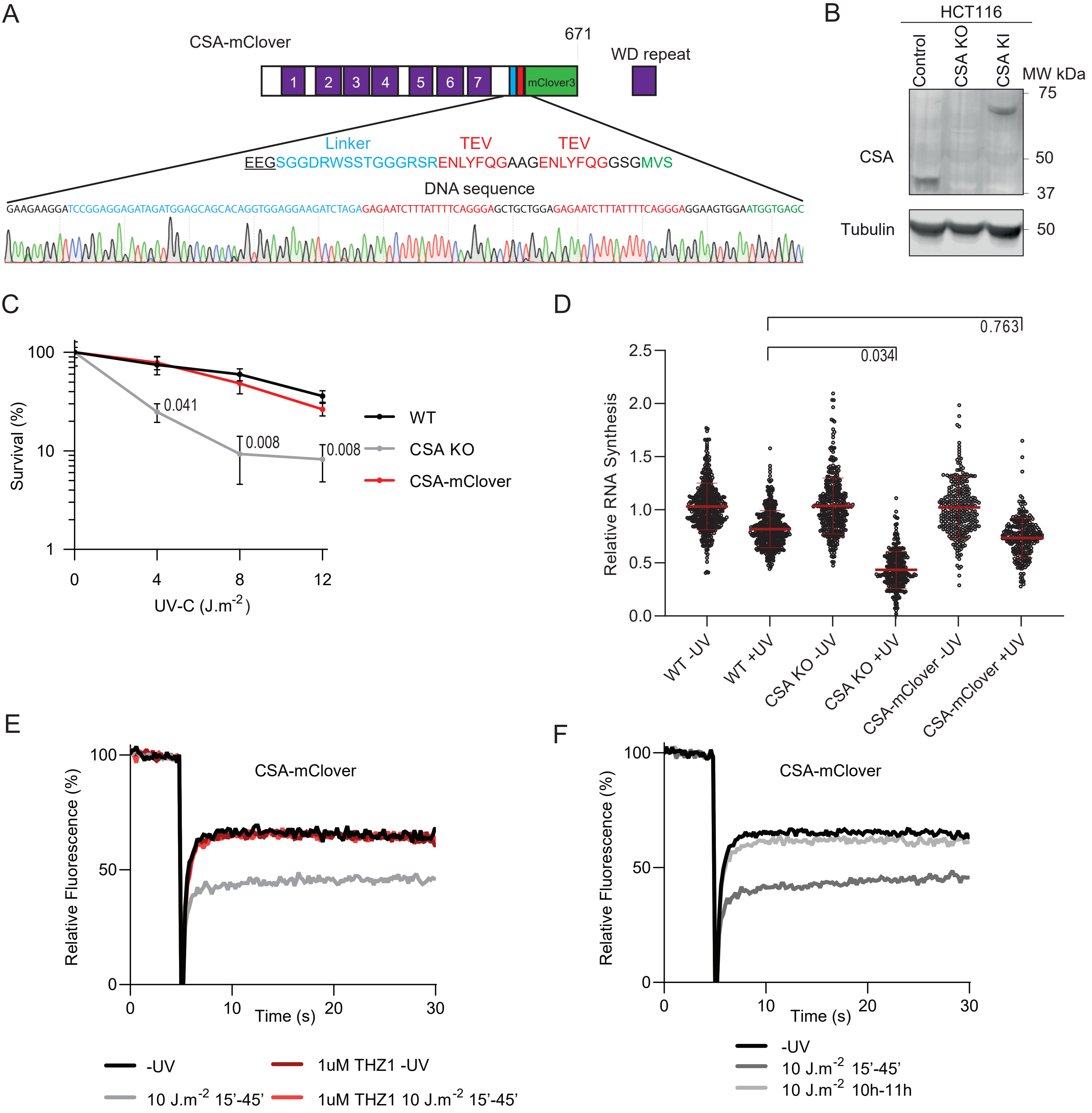

Supplementary Figure 1. Generation and characterization of CSA-mClover knock-in cells

A, Schematic representation of the CSA-mClover, which includes a linker and two TEV protease recognition sequences. **B**, Immunoblot of indicated HCT116 cell lines showing CSA or CSA-mClover expression. Tubulin was used as loading control. **C**, Relative colony survival of the indicated HCT116 WT, CSA-mC KI and CSAKO cells exposed to the indicated doses of UV. Graphs depict the mean ± SD from three independent experiments, the numbers represent p-values. p-values ≤ 0.05 were considered significant relative to WT analyzed by unpaired, two-tailed t-test, adjusted for multiple comparison. **D**, Transcription restart after UV damage as determined by relative EU incorporation in the indicated HCT116 WT, CSA-mC KI and CSAKO cells, at 24 h after UV exposure (10 J.m-²) or mock treated. RNA synthesis was measured by EU incorporation and levels were normalized to the non-irradiated cells (set to 1) and each normalized EU signal is shown as one data point. The mean ± S.D. is indicated in red from three independent experiments of (left to right) n=1149, 967, 926, 687, 756 and 718cells. **E,** Fluorescence Recovery After Photobleaching (FRAP) analysis of CSA-mClover mobility in presence or absence of THZ1inhibitor added 1 h before irradiation and followed by UV irradiation (10 J.m-²). Graphs depict the mean & S.E.M. of 30 cells for each condition from independent experiments. **F,** FRAP analysis of CSA-mClover in mock and UV treated (10 J.m-²) (the data are reported also in Supplementary Figure 15B). Graphs depict the mean & S.E.M. of 30 cells for each condition from three independent experiments. Data shown in **D**, numbers represent p-values (nested t-test, two-tailed). Source data are provided as a Source Data file.

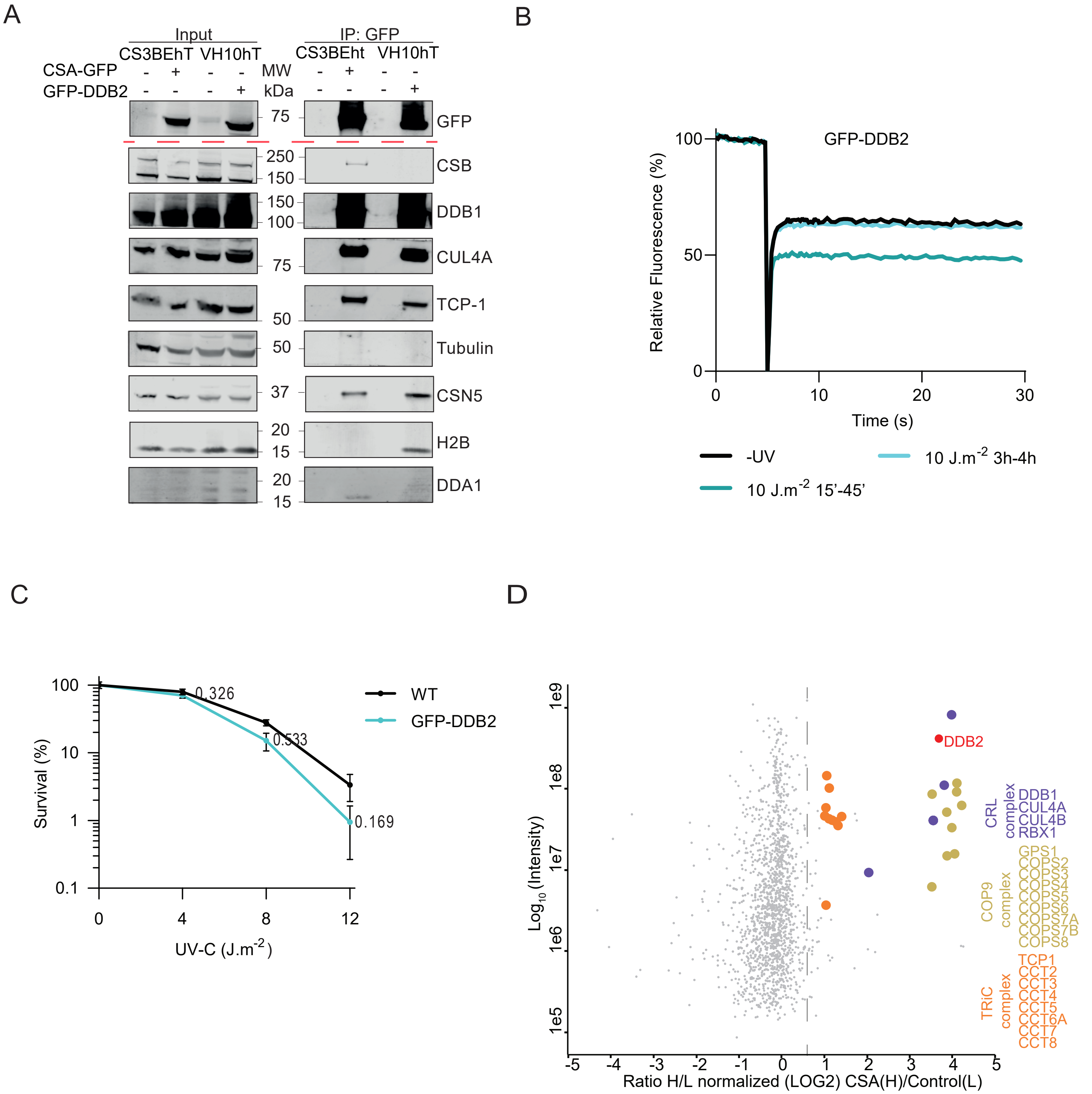

Supplementary Figure 2. Absence of DDA1 within CRL4^{DDB2}

A, IP of CSA and DDB2 using GFP beads from CSA-GFP expressed in CS3BEhT and GFP-DDB2 expressed in VH10hT cells followed by immunoblotting for the indicated proteins. CS3BEhT and VH10hT cells were used as a control. The experiment was repeated two times with similar results. **B**, FRAP analysis of GFP-DDB2 mobility from GFP-DDB2 KI HCT116 1 and 3 h after UV irradiation (10 J.m-²). Graphs depict the mean & S.E.M. of 30 cells for each condition from three independent experiments. **C**, Relative colony survival of the indicated HCT116 WT and GFP-DDB2 knock-in cells exposed to the indicated doses of UV. Graphs depict the mean ± SD from three independent experiments, the numbers represent p-values. p-values ≤ 0.05 were considered significant relative to WT analyzed by unpaired, two-tailed t-test, adjusted for multiple comparison. **D**, Scatter plot of Log_2 SILAC ratios of proteins isolated by GFP-pulldown from GFP-DDB2 KI HCT116 cells. The SILAC fold change (Log_2) is plotted on the x-axis and the signal intensity of the peptides is plotted on the y-axis. Source data are provided as a Source Data file.

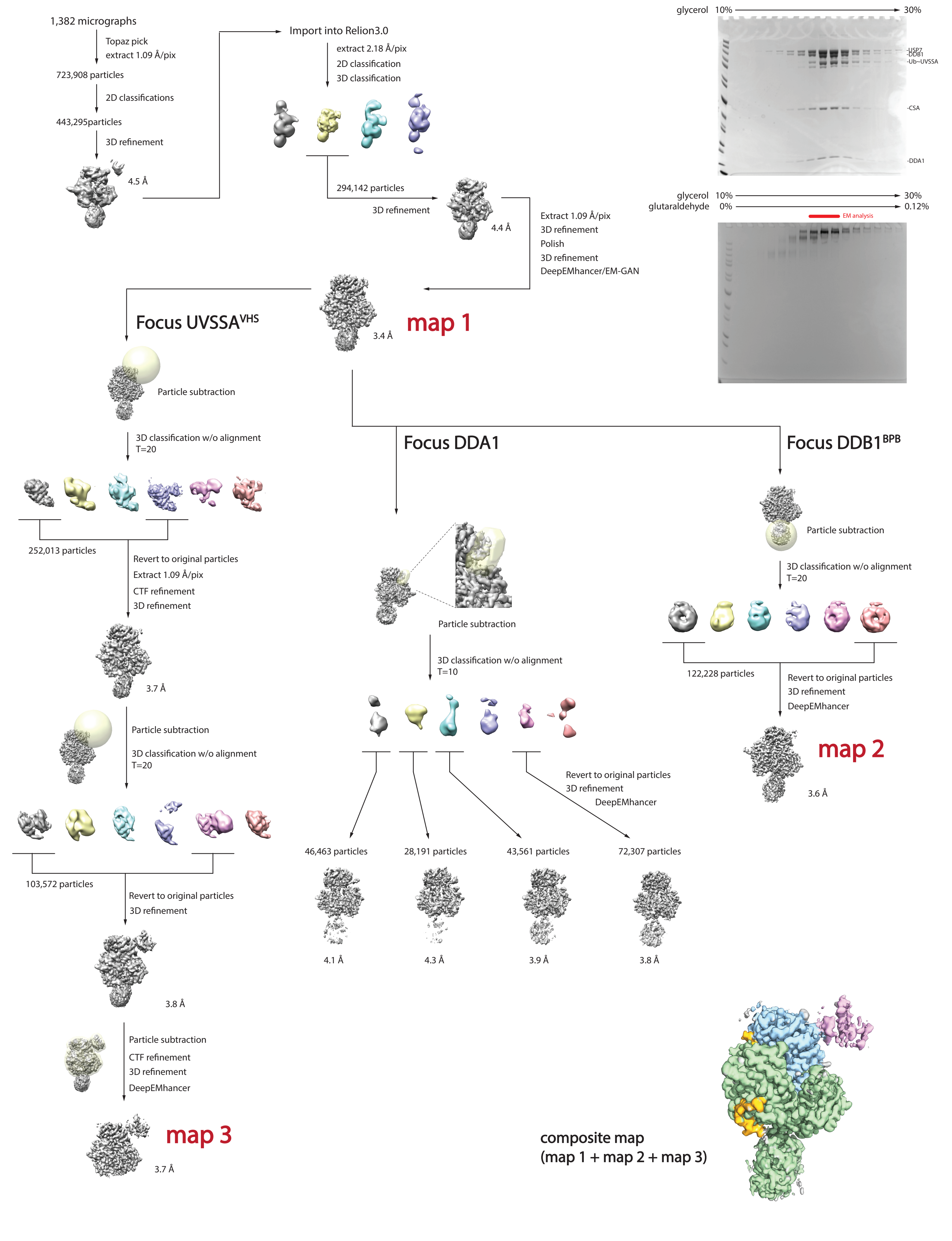

Supplementary Figure 3. Cryo-EM sample preparation and data processing
Reconstitution of USP7^{C223A}-Ub~UVSSA-CSA-DDB1-DDA1 in a glycerol gradient is shown in the right panel. For EM analysis, the complex is crosslinked with glutaraldehyde. The fractions used for cryo-EM analysis are highlighted in red. Steps in processing are as indicated. Structure was built in the composite map. Source data are provided as a Source Data file.

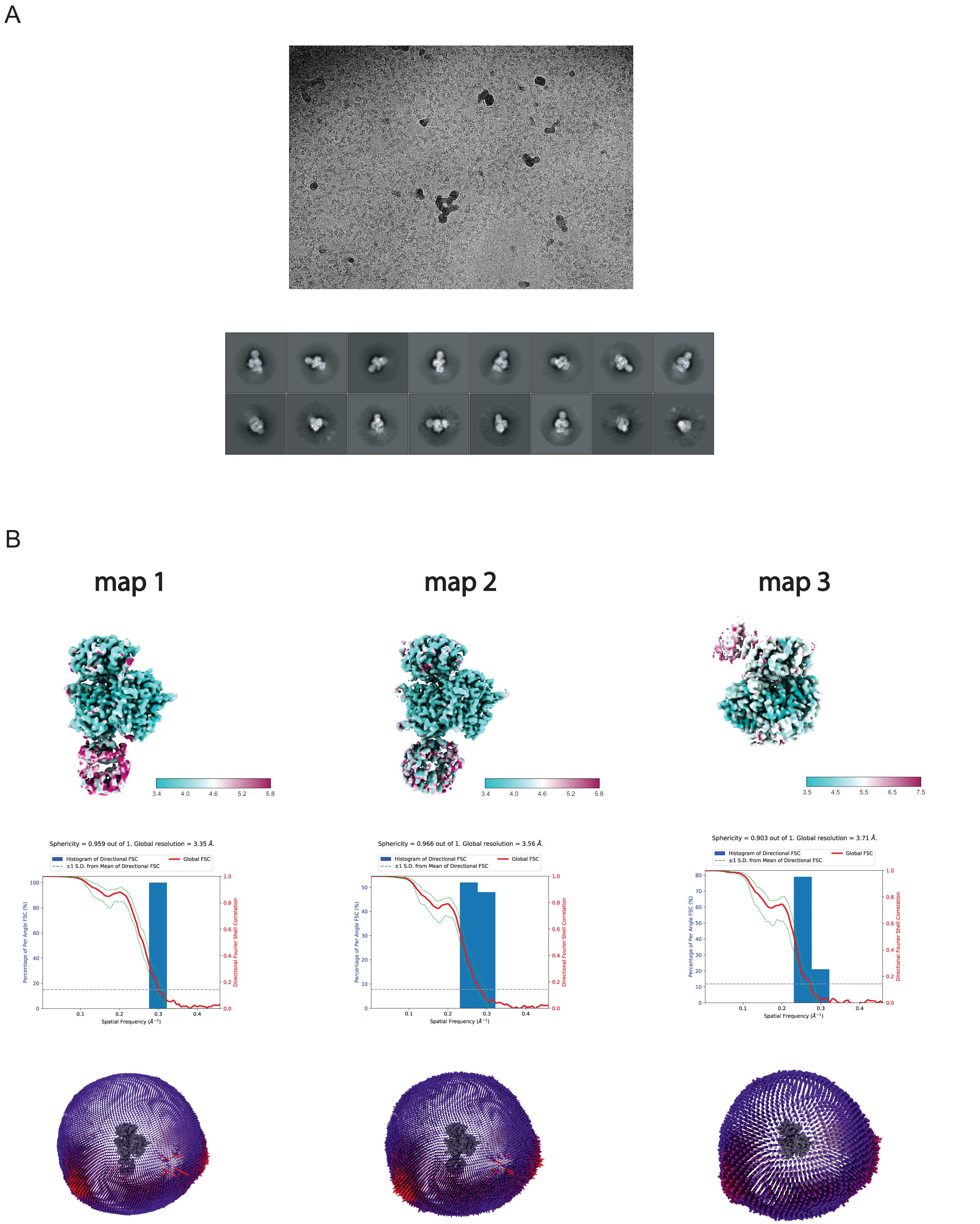

Supplementary Figure 4. Cryo-EM data validation

Cryo-EM data validation. **A**, Representative cryo-EM image is shown in the upper panel. Representative 2D classes are shown in the lower panel. **B**, Validation of the cryo-EM maps. In the upper panel, cryo-EM maps (see Supplementary Figure 3 for data processing) are colored in local resolution. The 3D FSC curves and angular distributions are shown in the middle and lower panel, respectively. Source data are provided as a Source Data file.

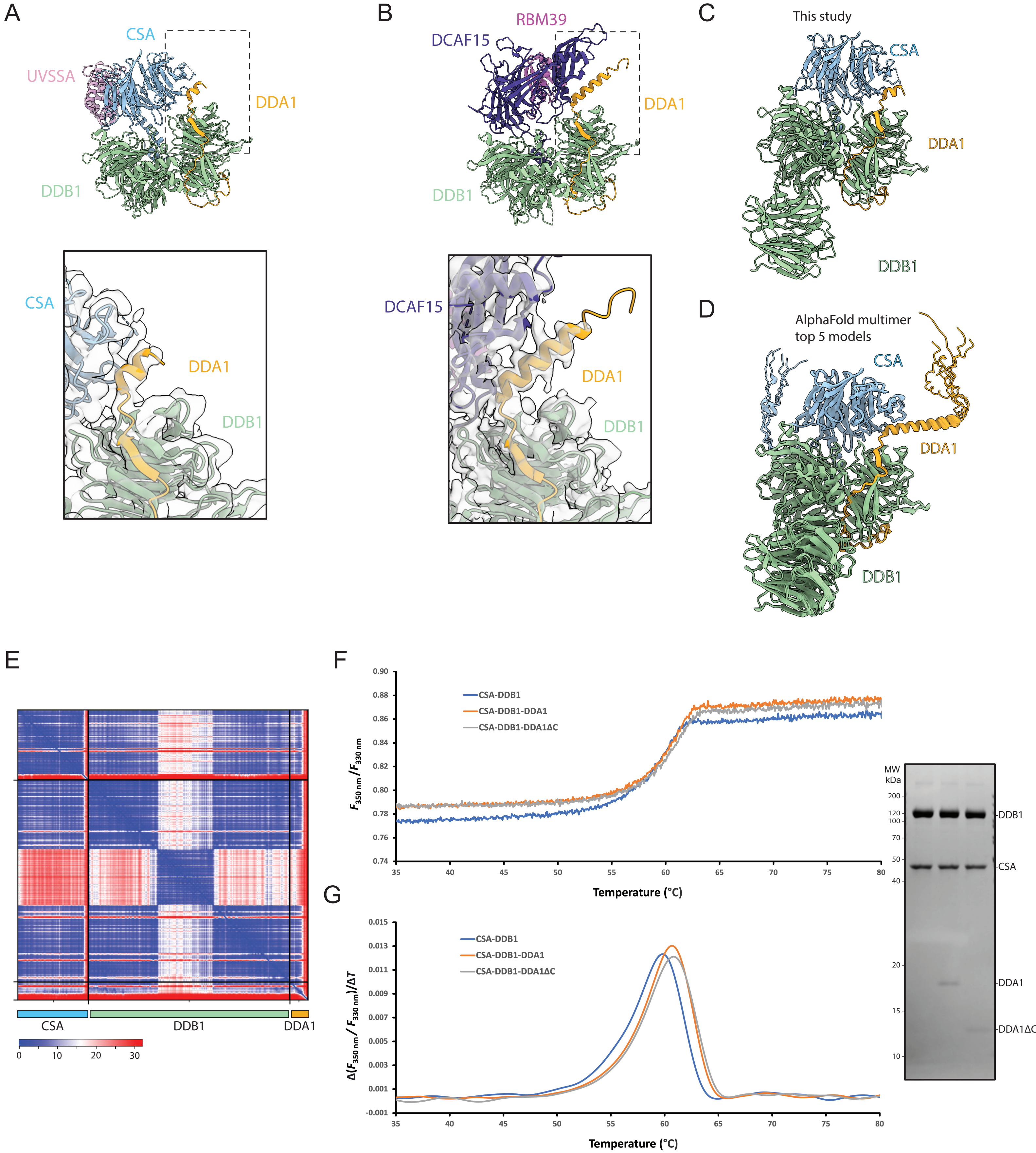

Supplementary Figure 5. Structure comparison

A-B, Structure comparison of DDA1-DCAF interactions. The UVSSA-CSA-DDB1-DDA1 structure (this study, panel **A**) is compared to RBM39-DCAF15-DDB1-DDA1 (PDB 6UD7, panel B). Close up views of DDA1-DCAF interactions are highlighted below with cryo-EM density. The atomic model 6UD7 is fitted into cryo-EM map (EMDB 10213). DCAF15 has an atypical β propeller configuration that the extended β sheet provides a large interaction interface with the DDA1 C-terminal helix. **C**, The atomic model of CSA-DDB1-DDA1 complex from this study. **D**, The top 5 models predicted by AlphaFold multimer were superimposed and shown in the same view as **C**. The predicted models are highly similar to the cryo-EM structure. **E**, The predicted aligned error (PAE) plot of the top 1 prediction. **F-G**, Thermostability analysis of CSA-DDB1 complexes. The stability of CSA-DDB1 was monitored by nanoDSF. **F,** The intrinsic fluorescence 350 nm/330 nm ratio of the samples. **G** The first derivative of the melting experiments. In the presence of DDA1 or DDA1Δ, the melting temperature increases about one degree. The experiment has been repeated multiple times and the results are reproducible. Source data are provided as a Source Data file.

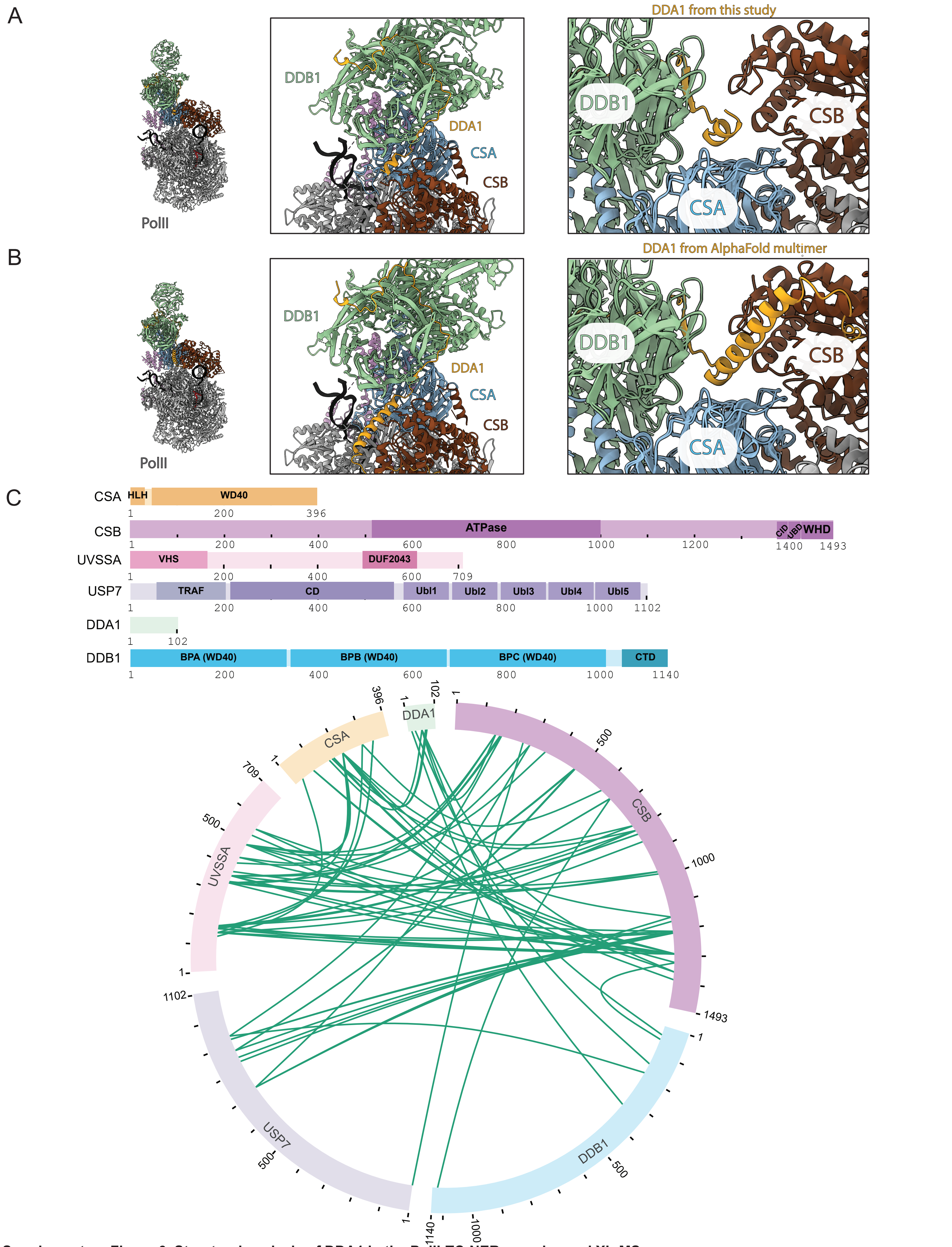

Supplementary Figure 6. Structural analysis of DDA1 in the PolII-TC-NER complex and XL-MS

A-B, Structural analysis of DDA1 in the PolII-TC-NER complex. **A**, The CSA-DDB1-DDA1 structure in this study is superimposed into the Pol II-TC-NER complex containing ELOF1 (PDB 8B3D). The C-terminal helix of DDA1 (in yellow) passes through a cavity created by CSA, DDB1 and CSB. **B**, The top 1 model of CSA-DDB1-DDA1 predicted by AlphaFold is superimposed into the Pol II-TC-NER complex as **A**. The extension of the DDA1 C-terminal helix passes through the cavity without clashing to other subunits of the Pol II complex. **C**, Cross-linking MS map of all identified residue linkages. Source data are provided as a Source Data file.

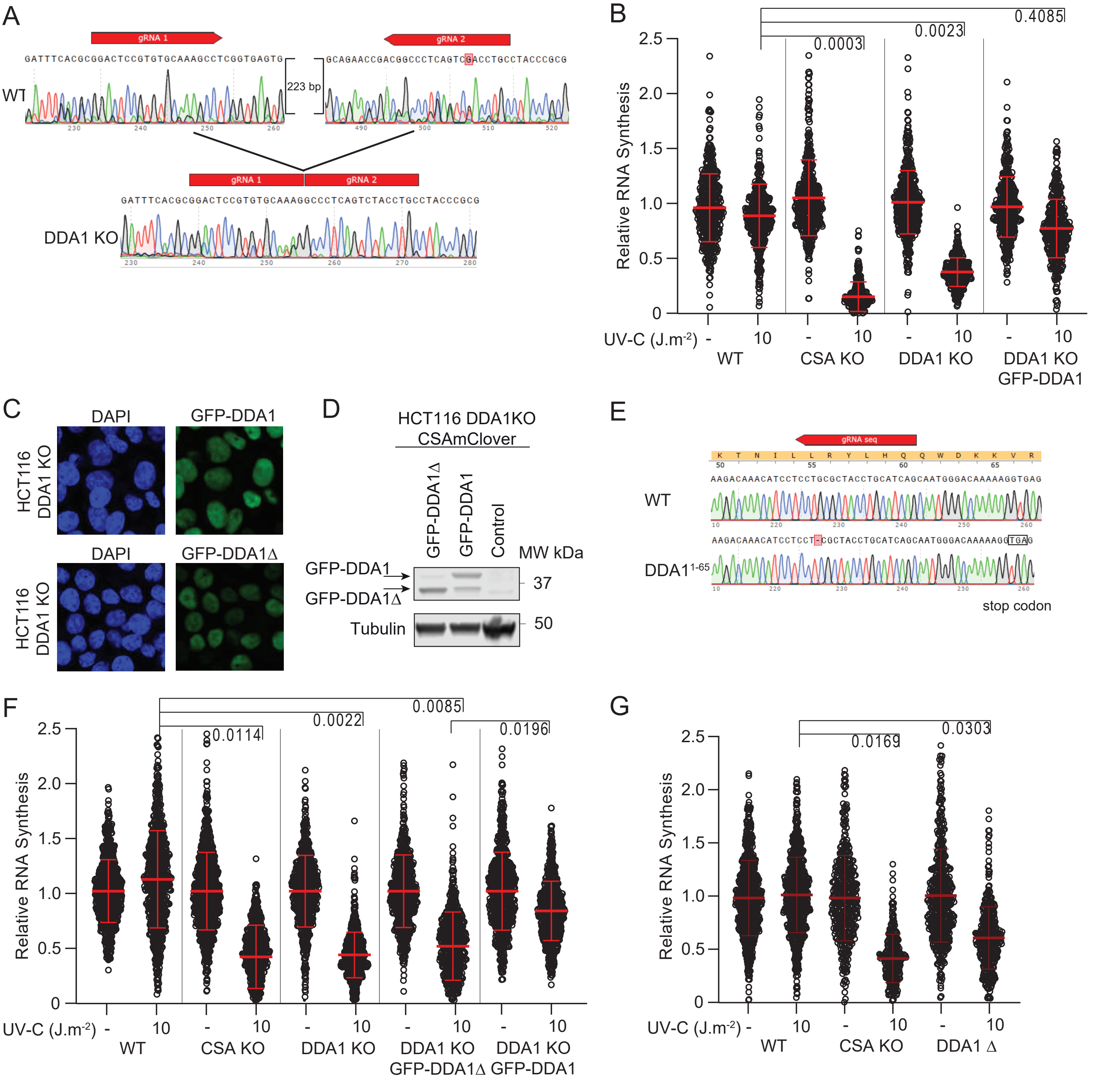

Supplementary Figure 7. DDA1 KO and DDA1 Δ

A, Sanger sequencing of the DDA1 locus exon 2 and 3 from HCT116 cells, showing deletion in the targeted genomic locus of DDA1. **B**, Transcription restart after UV damage as determined by relative EU incorporation in the indicated HCT116 WT and KO cells, with DDA1 re-expression where indicated, at 24 hours after UV exposure (10 J.m-²) or mock treated. RNA synthesis was measured by EU incorporation and levels were normalized to the non-irradiated cells (set to 1) and each normalized EU signal is shown as one data point. The mean ± S.D. is indicated in red from three independent experiments of (left to right) n=931, 1026, 1004, 638, 1122, 839, 703 and 593 cells. **C**, Representative immunofluorescence images of GFP-DDA1 and GFP-DDA1Δ in DDA1KO HCT116 cells. **D**, Immunoblot of cell extracts from the HCT116 DDA1KO cells transiently transfect, stained for the indicated proteins. Tubulin was used as loading control. **E**, Sanger sequencing of the DDA1 locus exon 4 from HCT116 cells, showing frameshift mutations (stop codon) in the targeted genomic locus of DDA1. **F**, Transcription restart after UV damage as determined by relative EU incorporation in the indicated HCT116 WT and KO cells, with DDA1 and DDA1Δ re-expression where indicated, at 24 hours after UV exposure (10 J.m-²) or mock treated. RNA synthesis was measured by EU incorporation and levels were normalized to the non-irradiated cells (set to 1) and each normalized EU signal is shown as one data point. The mean ± S.D. is indicated in red from three independent experiments of (left to right) n=779, 717, 962, 592, 661, 634, 656, 605, 719 and 612 cells. **G**, Transcription restart after UV damage as determined by relative EU incorporation in the indicated CSA-mClover, CSA-mClover DDA11-65 and CSAKO HCT116 cells at 24 hours after UV exposure (10 J.m-²) or mock treated. RNA synthesis was measured by EU incorporation and levels were normalized to the non-irradiated cells (set to 1) and each normalized EU signal is shown as one data point. The mean ± S.D. is indicated in red from two independent experiments of (left to right) n=694, 735, 436, 297, 503 and 382 cells. Data shown in **B**, **F** and **G**, numbers represent p-values (nested t-test, two-tailed). Source data are provided as a Source Data file.

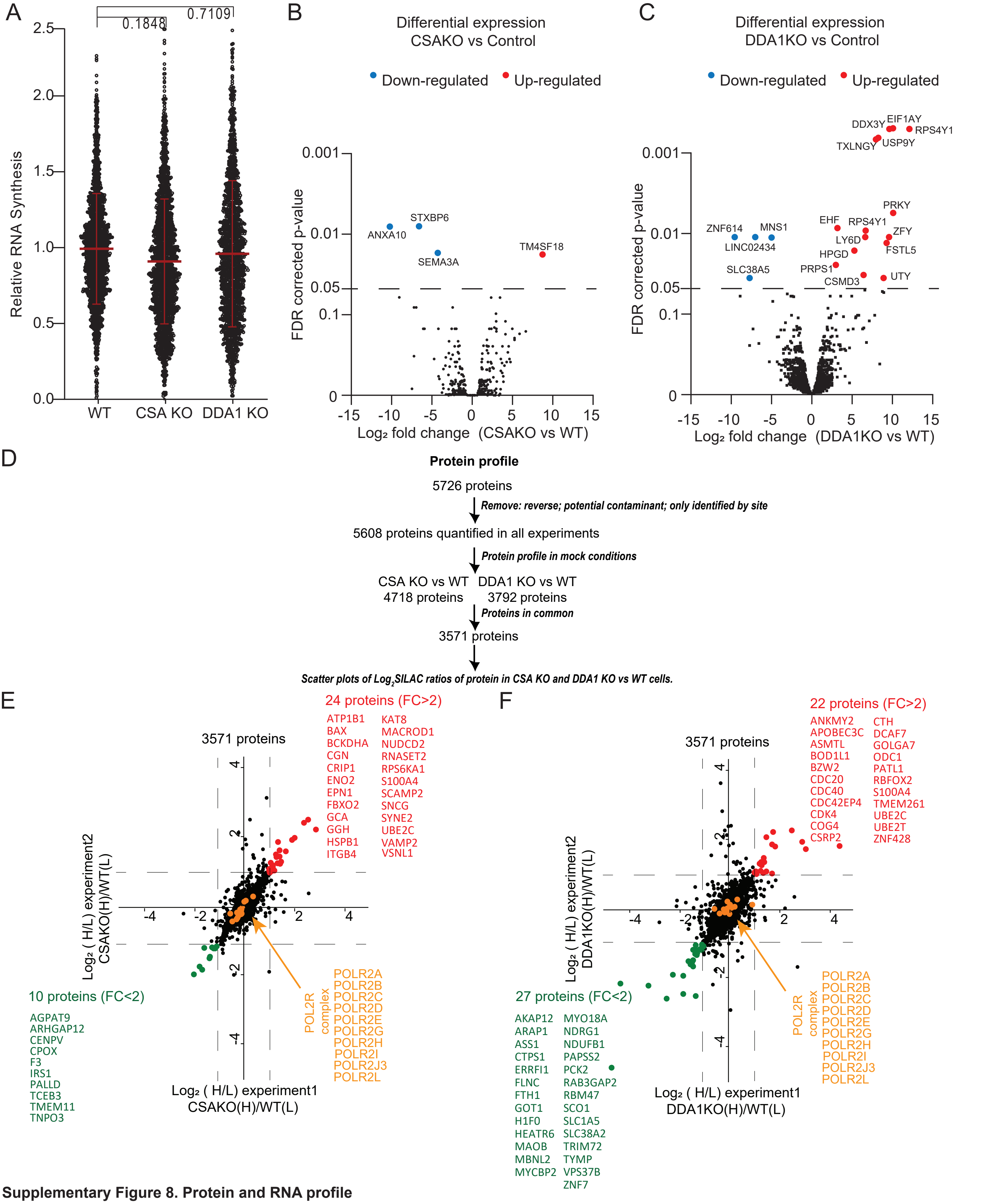

Supplementary Figure 8. Protein and RNA profile

A, RNA synthesis in non-irradiated cells (Figure 3 B, D) as determined by relative EU incorporation in the indicated HCT116 WT and KO cells. RNA synthesis was measured by EU incorporation and levels were normalized to the HCT116 WT cells (set to 1) and each normalized EU signal is shown as one data point. The mean ± S.D. is indicated in red from six independent experiments of (left to right) n=1628, 1781 and 1474 cells. **B**, Volcano plot of differentially expressed genes between WT and CSAKO HCT116 cells. Each red/green dot represents a significantly differentially down-regulated or up-regulated gene. **C**, Volcano plot of differentially expressed genes between WT and DDA1KO HCT116 cells. Each red/green dot represents a significantly differentially down-regulated or up-regulated gene. **D**, Experimental set up and step-by-step proteomics workflow to obtain the global protein profile. **E-F,** Scatter plots of Log_2SILAC ratios of protein in HCT116 WT, CSAKO, DDA1KO cells. The experiments were conducted in duplicate comparing WT mock-treated versus CSA and DDA1KO mock-treated HCT116 cells (**E-F**). Data shown in **A**, numbers represent p-values (nested t-test, two-tailed). Differentially expression shown in **B** and **C** was analyzed using the glmQLFTest. P-values were corrected using Benjamini-Hochberg procedure. Any genes with thus calculated false discovery rate not surpassing 0.05 were deemed statistically significantly differentially expressed. Source data are provided as a Source Data file.

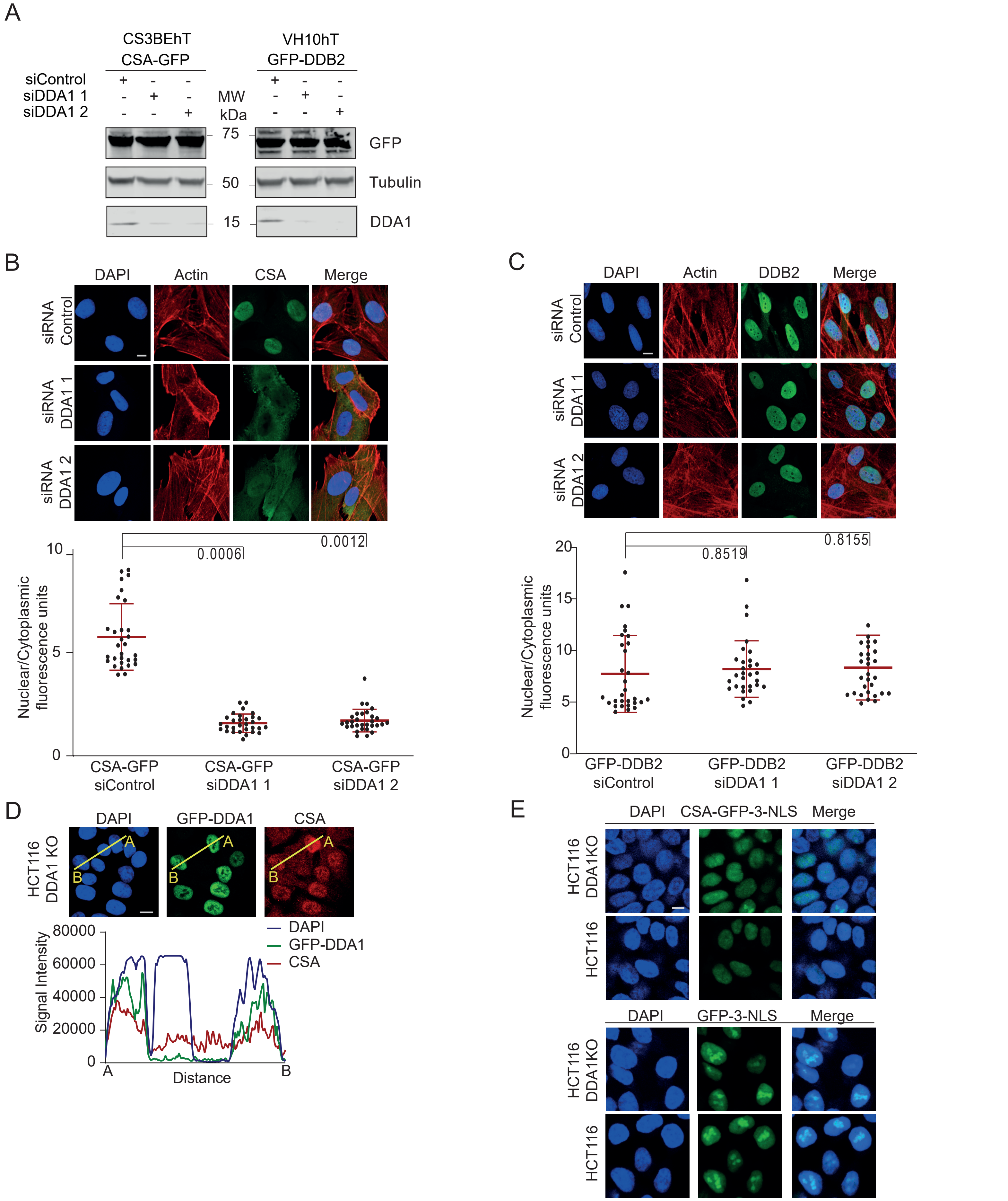

Supplementary Figure 9. CSA and DDB2 nuclear localization

A, Immunoblot showing endogenous CSA-GFP, GFP-DDB2 and DDA1 levels in the indicated cell lines. Tubulin and H2B were used as loading control. **B, C** Representative immunofluorescence images of CSA-GFP and GFP-DDB2 expressed in CS3BEhT and VH10hT cells, respectively scale bar: 10 µm. Graphs below: Nuclear over cytoplasmic ratios of CSA and DDB2 levels in CS3BEhT and VH10hT cells, respectively, were analyzed and quantified by fluorescence microscopy and ImageJ. The mean ± S.D. is indicated in red from three independent experiments of (left to right) n=30, 30, 30, 30, 30 and 30 images. CSA and DDB2 signal intensity at nucleus (as identified by DAPI staining) was compared to that in the rest of the cell (phalloidin). **D**, Representative immunofluorescence images of endogenous CSA in GFP-DDA1 re-expression HCT116 DDA1KO cells. The dashed lines indicate the line-scan track used to quantify fluorescence intensity of CSA (red) and GFP-DDA1 (green). The nucleus was identified using DAPI staining (blue). **E**, Representative immunofluorescence images of CSA-GFP-3NLS and GFP-3NLS in WT and DDA1KO HCT116 cells, scale bar: 10 µm. Data shown in **B** and **C**, numbers represent p-values (nested t-test, two-tailed). Source data are provided as a Source Data file.

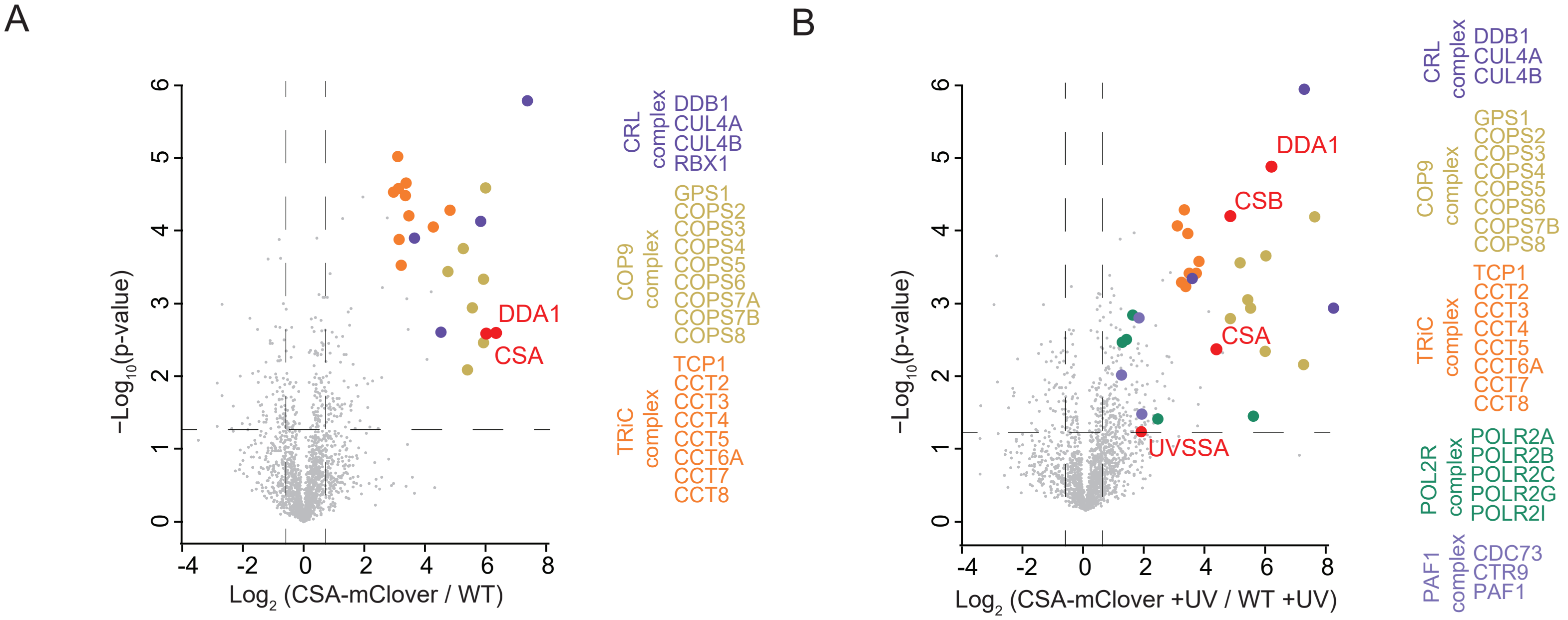

C

			CSAmClover/DDA1KO vs CSAmClover<br>t-test Difference (Log₂)	CSAmClover/DDA1KO +UV vs CSAmClover +UV<br>t-test Difference (Log₂)
	CSA (ERCC8)	→	**-0.175833**	**0.594915**
	DDB1	→	0.384607	0.464457
	DDA1	↓	-3.23592	-3.68452
TRiC complex	TCP1	→	0.211027	0.454005
	CCT2	→	0.190139	0.436647
	CCT3	→	0.215641	0.336879
	CCT4	→	0.121384	0.393906
	CCT5	→	0.222755	0.31822
	CCT6A	→	0.220267	0.274117
	CCT7	→	0.251577	0.372348
	CCT8	→	0.1789	0.344252

Supplementary Figure 10. CSA's protein partners

A-B Volcano plots depicting the statistical differences between three replicates of the MS analysis after GFP immunoprecipitation of CSA-mClover mock-treated (**A**) or UV treated (**B**) in HCT116 cells. The fold change (log_2) is plotted on the x-axis and the significance (t-test $-Log_{10}$(p value), Two-sample test, two-tailed) is plotted on the y-axis. RNAPII subunits are indicated in green, PAF1 subunits in light purple, TRiC subunits in orange, COP9 subunits in yellow, CRL subunits in dark purple and TC-NER factors in red. **C**, Quantitative interaction proteomics of CSA-mClover from CSA-mC KI DDA1KO cells relative to CSA-mC KI WT cells. Reduced and no changed interactions in absence of DDA1 are indicated by red and yellow arrows respectively. Source data are provided as a Source Data file.

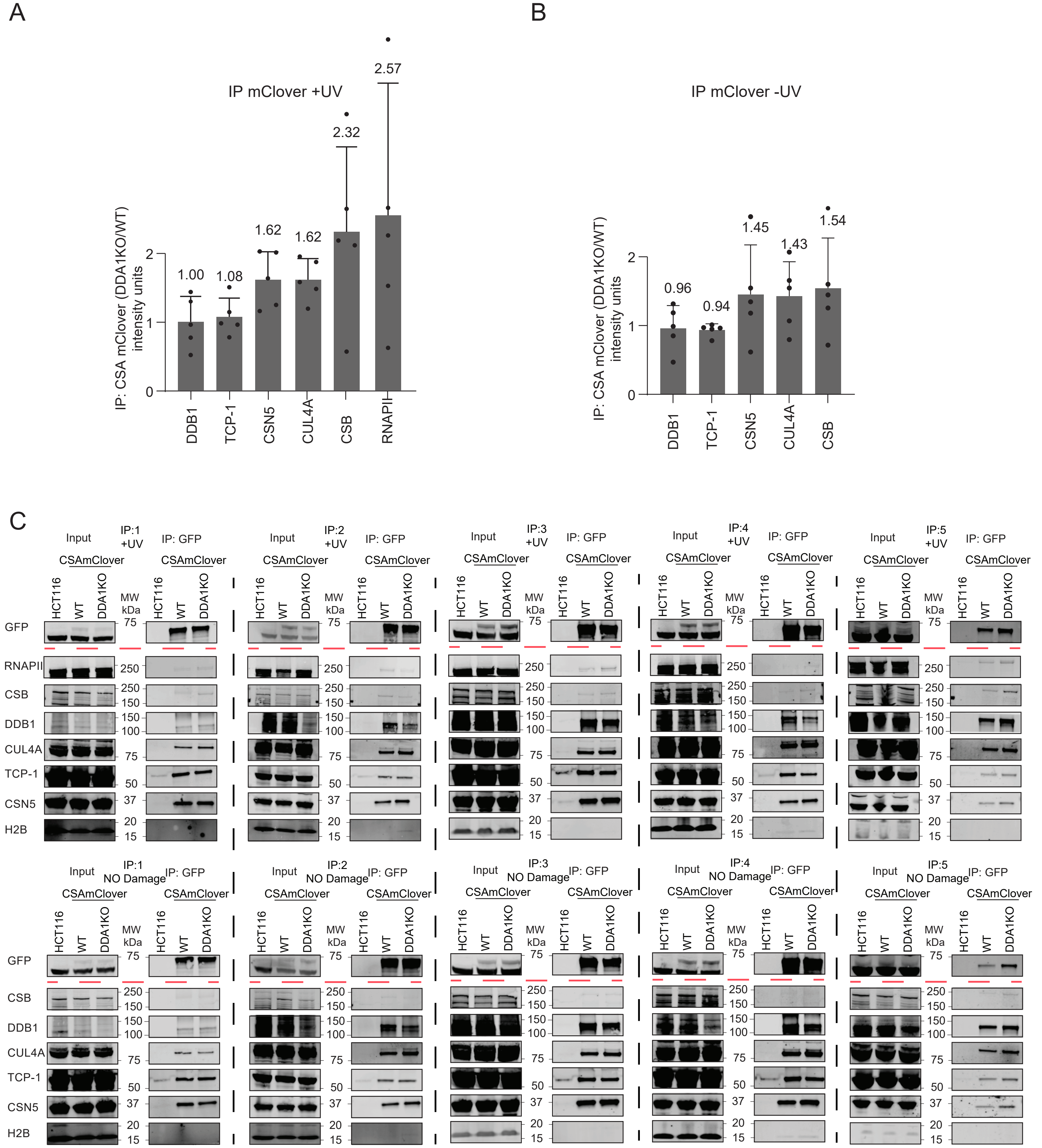

Supplementary Figure 11. IP CSA-mClover

A-B Quantification of the immunoblot analysis (IP CSA-mClover). The band intensities of the indicated proteins were normalized to GFP signal and expressed as ratio between DDA1KO and WT cells in mock and UV treated (n = 5 experiments), set at 1. **C**, IP of CSA-mClover using GFP beads in CSA-mC KI and CSA-mC DDA1KO HCT116 cells followed by immunoblotting for the indicated proteins. HCT116 cells were used as a control. Source data are provided as a Source Data file.

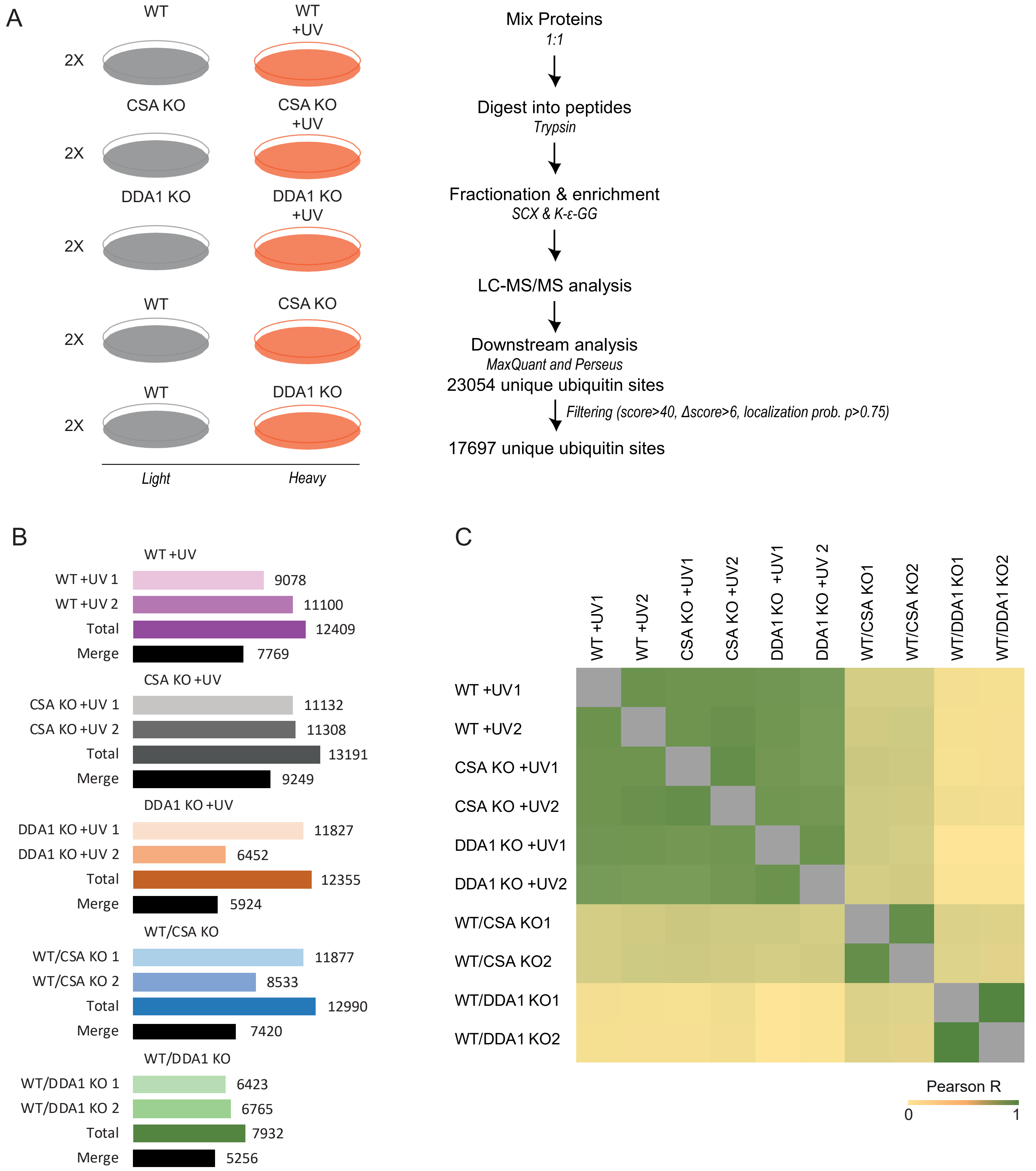

Supplementary Figure 12. Ubiquitin profile

A, Experimental set up and step-by-step proteomics workflow to obtain the global ubiquitin profile. **B**, Coverage of the global proteome indicating the number of ubiquitinated peptides quantified for each experiment. **C**, Heatmap shows Pearson R values of Log_2 ubiquitinated peptides SILAC ratios. Source data are provided as a Source Data file.

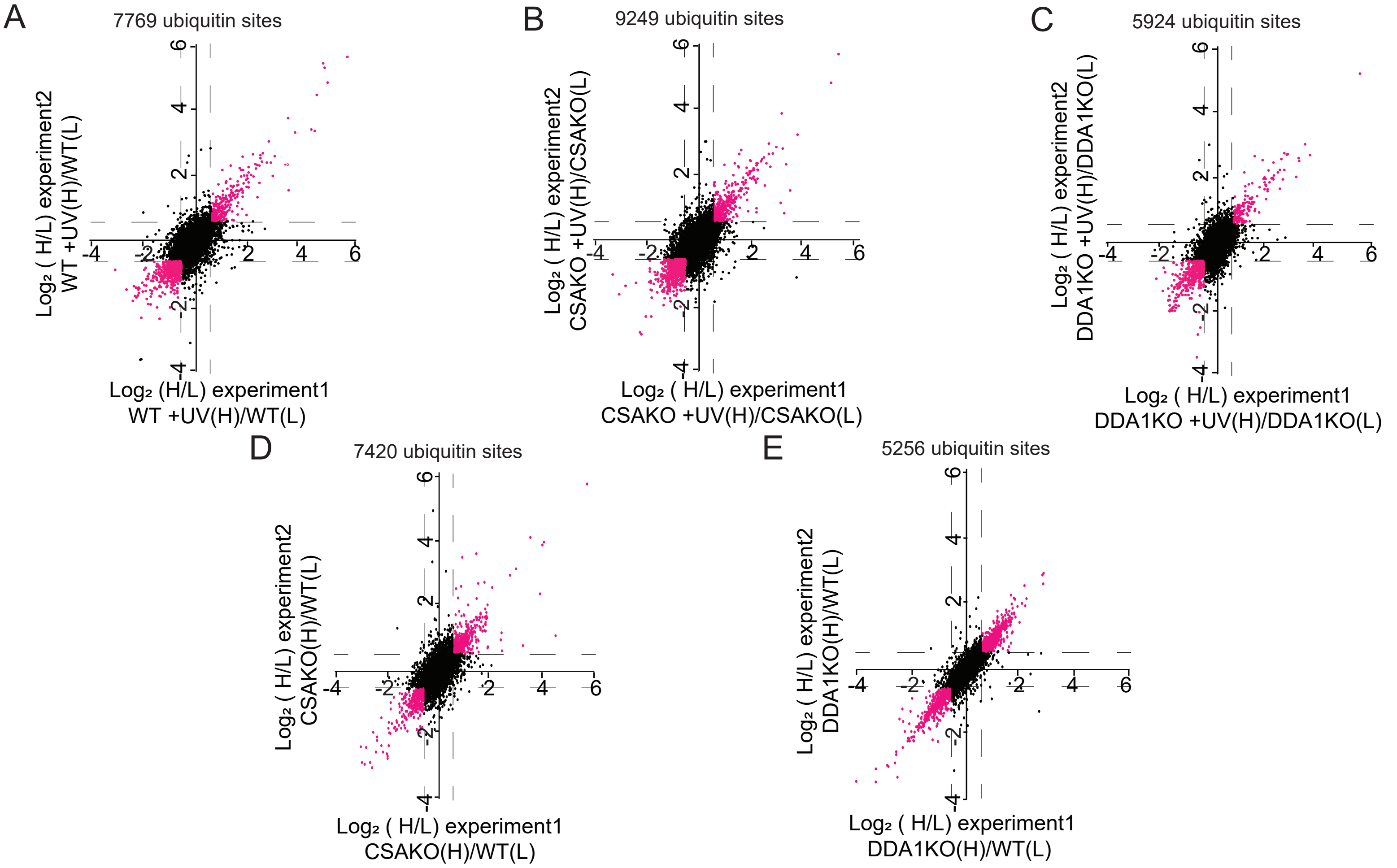

Supplementary Figure 13. Scatter plots ubiquitin profile

A-E, Scatter plots of Log₂SILAC ratios of ubiquitin sites in HCT116 WT, CSAKO, DDA1KO cells. The experiments were conducted in duplicate comparing mock-treated versus UV-treated cells (**A-C**) or WT mock-treated versus KOs mock-treated HCT116 cells (**D-E**). Source data are provided as a Source Data file.

A

Top 50 up-regulated ubiquitin sites (WT)

GENE NAME	Ratio H/L (Log_2)	
(position)	Experiment1	Experiment2
XPC (364)	5.65	5.58
ERCC2 (751)	4.91	4.80
SF3B1 (1014)	4.80	5.26
XPC (374)	4.75	5.39
XPC (359)	4.50	4.43
HELLS (204)	4.42	3.32
TAP2 (245)	4.30	3.37
TRIP4 (245)	3.70	3.29
PSMA2 (171)	3.45	1.53
PSMC1 (413)	3.43	3.72
TMA7 (30)	3.40	2.31
HSPA8 (500)	2.93	2.56
YBX1 (118)	2.86	1.96
POLR2A (1268)	2.78	2.37
ACLY (1077)	2.71	3.01
POLR2A (1350)	2.64	2.36
SMARCA5 (814)	2.50	2.65
DDB2 (309)	2.45	2.40
XPC (174)	2.40	2.60
DDB2 (362)	2.39	2.51
OLA1 (190)	2.35	2.27
MYO10 (918)	2.35	1.75
CNBP (103)	2.28	2.29
RPS16 (4)	2.22	2.18
RPS12 (129)	2.22	2.32
CUEDC2 (272)	2.14	1.95
YBX3 (96)	2.12	2.62
YBX1 (64)	2.12	2.62
SND1 (513)	2.04	1.99
RPS27A (152)	1.96	2.06
RPL12 (48)	1.95	2.22
USP48 (551)	1.95	1.77
SUPT6H (306)	1.94	1.62
POLR2A (177)	1.91	1.89
RPS3 (230)	1.89	1.97
ABCE1 (397)	1.89	1.63
RPS10 (139)	1.85	1.84
XPC (161)	1.79	1.98
CNBP (8)	1.78	1.74
TIA1 (79)	1.74	2.14
UVSSA (414)	1.74	2.55
TOP1 (642)	1.73	1.50
PHRF1 (556)	1.70	1.57
CSDE1 (682)	1.68	2.14
ASCC3 (198)	1.67	1.95
CSDE1 (288)	1.67	1.53
RNH1 (46)	1.62	2.20
PHRF1 (768)	1.56	1.69
RNF170 (3)	1.56	1.50
CLPTM1 (460)	1.55	1.79

B

Top 50 up-regulated ubiquitin sites (CSA KO)

GENE NAME	Ratio H/L (Log_2)	
(position)	Experiment1	Experiment2
SF3B1 (1014)	5.42	5.68
XPC (374)	5.12	4.82
DSP (1033)	3.81	3.23
SH2B3 (373)	3.53	1.52
HSPA8 (500)	3.38	2.33
YBX1 (118)	3.25	2.81
PSMC1 (413)	3.21	3.88
DDB2 (309)	3.00	2.85
SMARCA5 (814)	2.86	2.65
CNBP (103)	2.77	2.25
DDB2 (106)	2.72	2.64
XPC (174)	2.71	2.96
SUPT6H (306)	2.61	2.36
SIVA1 (41)	2.57	1.68
OLA1 (190)	2.53	2.53
YBX3 (96)	2.44	2.48
YBX1 (64)	2.44	2.48
POLR2A (1350)	2.41	2.41
EDF1 (98)	2.28	2.15
RPS16 (4)	2.27	2.17
PRR14L (1861)	2.26	1.89
USP48 (551)	2.26	2.36
SUPT5H (258)	2.24	2.29
RPS12 (129)	2.24	2.36
MYH9 (856)	2.21	1.78
SPATS2L (11)	2.19	1.83
CNBP (8)	2.15	1.86
RPS10 (139)	2.14	2.16
SND1 (513)	2.12	2.21
XPC (161)	2.12	1.95
POLR2A (163)	2.09	2.15
POLR2A (1268)	2.09	1.90
CSDE1 (682)	2.03	1.88
RPL12 (48)	2.02	2.11
ACLY (1077)	2.01	2.43
ASCC3 (78)	1.99	2.44
RPS19 (111)	1.99	1.90
EIF2S2 (276)	1.96	2.04
BAZ2A (687)	1.96	2.11
POLR2A (177)	1.96	2.07
RPS3 (230)	1.91	2.00
PLEC (3106)	1.91	1.59
CUEDC2 (272)	1.86	2.06
POLH (709)	1.85	2.23
DCBLD2 (391)	1.82	1.85
TOP1 (642)	1.81	1.77
TOP1 (347)	1.80	1.61
SYNCRIP (297)	1.74	1.70
PHRF1 (768)	1.73	1.76
ASCC3 (213)	1.73	1.92

C

Top 50 up-regulated ubiquitin sites (DDA1 KO)

GENE NAME	Ratio H/L (Log_2)	
(position)	Experiment1	Experiment2
SF3B1 (1014)	5.21	5.16
PSMC1 (413)	3.38	2.68
TAP2 (245)	3.21	3.02
SMARCA5 (814)	2.99	2.51
XPC (183)	2.97	2.59
EDRF1 (694)	2.88	2.02
POLR2A (1268)	2.57	2.71
XPC (174)	2.53	2.70
POLR2A (1350)	2.44	2.24
OLA1 (190)	2.44	2.75
HSPA8 (500)	2.38	1.95
YBX3 (93)	2.33	2.57
YBX1 (64)	2.33	2.57
RPL12 (48)	2.28	2.24
ARIH1 (293)	2.27	1.66
RNF219 (502)	2.20	2.15
CSDE1 (682)	2.07	2.07
RPS10 (139)	2.05	1.96
SYNCRIP (407)	2.04	2.46
POLR2A (177)	2.00	1.99
DDB2 (106)	1.96	1.97
XPC (161)	1.90	1.85
USP48 (551)	1.86	2.22
POLR2A (163)	1.83	1.65
FASN (1752)	1.82	1.59
CNBP (8)	1.78	1.35
PHRF1 (556)	1.78	1.75
RPS3 (230)	1.75	1.79
RPS16 (4)	1.75	1.96
GAR1 (150)	1.72	1.65
YBX3 (150)	1.72	2.00
CSDE1 (288)	1.72	1.85
SUPT6H (306)	1.70	1.97
TMA7 (30)	1.68	1.96
UVSSA (414)	1.64	2.54
RPS2 (58)	1.53	1.71
ABCE1 (397)	1.45	1.44
ASCC3 (319)	1.37	1.04
NUMA1 (1475)	1.36	2.65
DDB1 (204)	1.33	1.25
H1F0 (12)	1.28	1.19
RPS27A (113)	1.26	1.39
SMARCA5 (836)	1.26	1.26
SUPT6H (1100)	1.26	1.83
DRG1 (46)	1.25	1.19
PSIP1 (6)	1.24	1.39
RPS3 (214)	1.23	1.34
TANK (199)	1.23	1.19
RPA1 (167)	1.21	1.11
SND1 (886)	1.17	1.36

Supplementary Figure 14. Top 50 up-regulated ubiquitin sites

A-C, top 50 up-regulated ubiquitin sites in WT (**A**), CSAKO (**B**), DDA1KO (**C**) HCT116 cells treated with UV irradiation in both experiments. Source data are provided as a Source Data file.

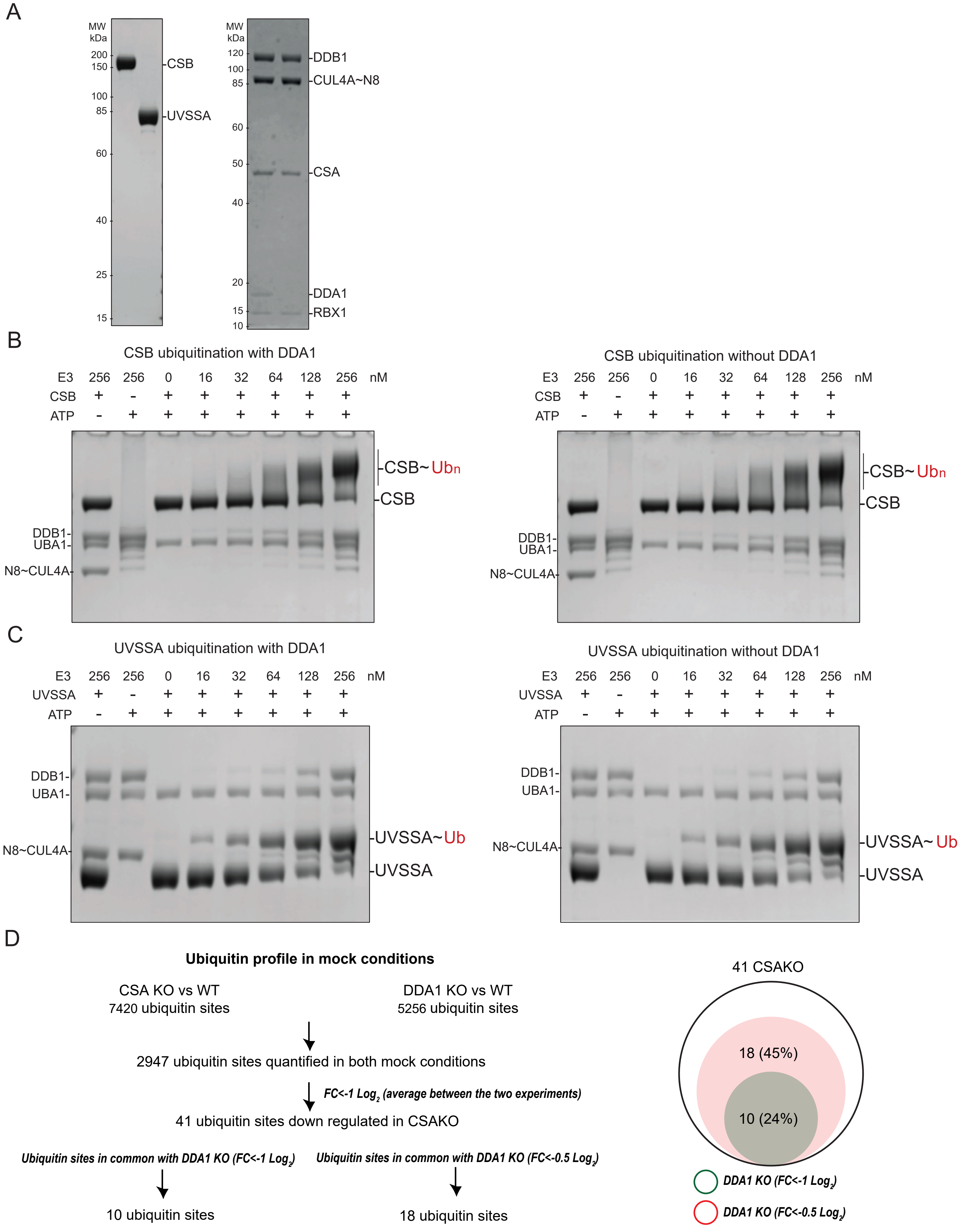

Supplementary Figure 15. Ubiquitin in vitro assays

A, Purified proteins for in vitro assays. **B**, In vitro ubiquitination assay of CSB in the presence and absence of DDA1. The reactions were mediated by the E2 enzyme UBE2D3 (UbcH5c). The experiment was repeated two times with similar results. **C**, In vitro ubiquitination assay of UVSSA in the presence and absence of DDA1. The reactions were mediated by the E2 enzyme UBE2E1 (UbcH6). Reactions were analyzed by SDS-PAGE and Coomassie blue staining. The results show that DDA1 has no effect on E3 ligase activity. The experiment was repeated two times with similar results. **D**, Experimental set up and step-by-step proteomics workflow to obtain the comparison of ubiquitin sites profile between CSA and DDA1KO HCT116 cells. Source data are provided as a Source Data file.

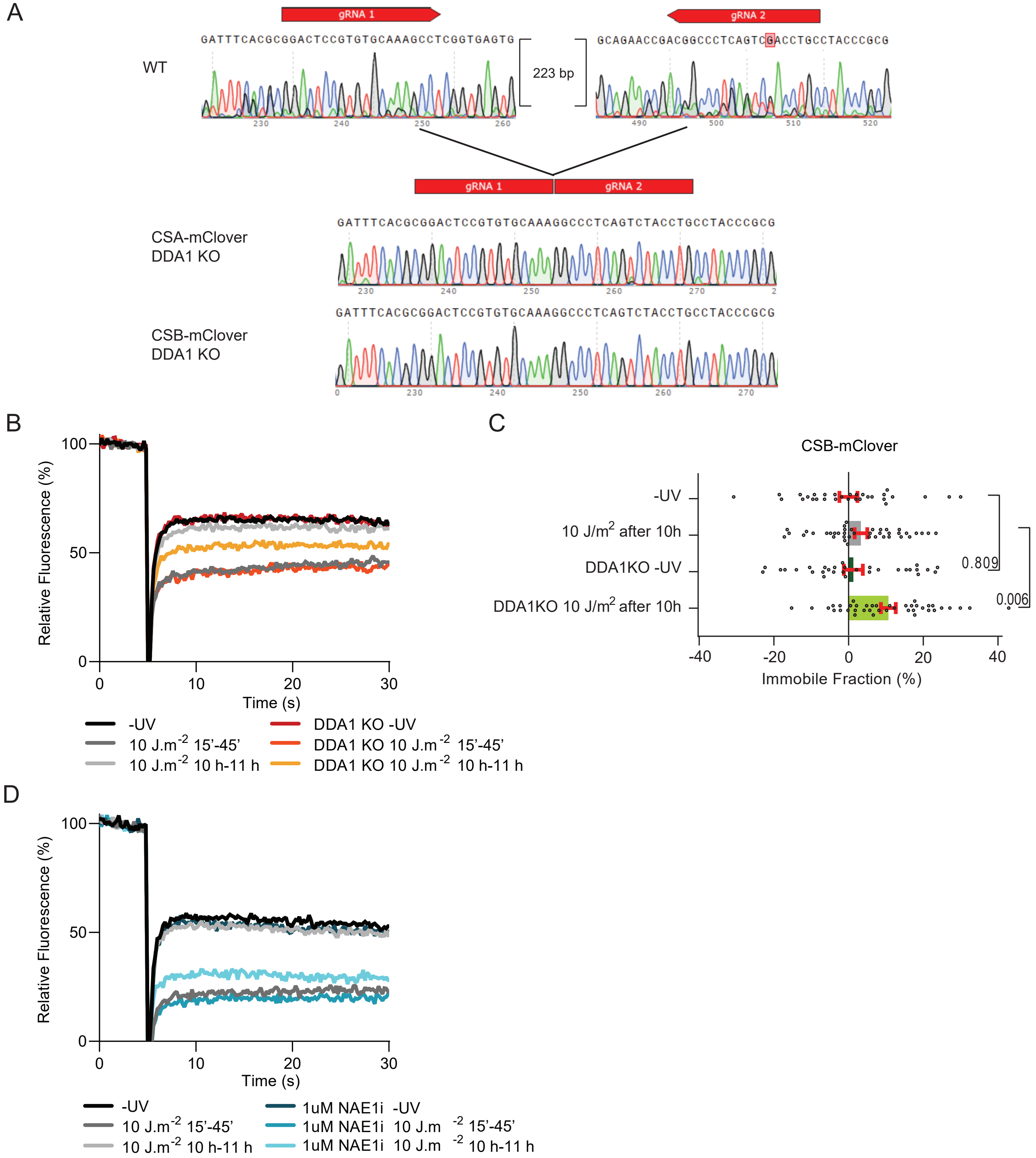

Supplementary Figure 16. FRAP analysis

A, Sanger sequencing of the DDA1 locus exon 2 and 3 from HCT116 cells, showing deletion in the targeted genomic locus of DDA1. **B**, FRAP analysis of CSA-mClover in mock and UV treated (10 J.m-²) using WT (the data are reported also in Supplementary Figure 1F) or DDA1KO cell lines. Graphs depict the mean & S.E.M. of 30 cells for each condition from three independent experiments. **C**, FRAP analysis of CSB-mClover in mock or UV irradiated (10 J.m-²) HCT116 WT and DDA1KO cells, measured at the indicated time points. Percentage of CSB-mClover immobile fraction was determined from FRAP analyses. Graphs depict the mean & S.E.M. of (top to bottom) n=30, 40, 30 and 40 cells from at least three independent experiments. **D**, FRAP analysis of CSA-mClover in presence or absence of NAEi added 0.5 h before irradiation and followed by UV irradiation (10 J.m-²). Graphs depict the mean & S.E.M. of 30 cells for each condition from three independent experiments. Data shown in **C**, numbers represent p-values (unpaired, two-tailed t-test adjusted for multiple comparisons). Source Data are provided as a Source Data file.

Cryo-EM data collection, refinement and validation statistics

	CSA-DDB1-DDA1 (Map 1) (EMDB-18377)	CSA-DDB1-DDA1 (Map 2) (EMDB-18378)	CSA-DDB1$^{BPA/BPC}$-DDA1-UVSSA^{VHS} (Map 3) (EMDB-18380)	CSA-DDB1-DDA1-UVSSA^{VHS} (composite map) (EMDB-18398) (PDB-8QH5)
**Data collection and processing**				
Magnification			81 000×	
Voltage (kV)			300	
Electron exposure (e–/Å^2)			60	
Defocus range (μm)			-1.0 to -3.0	
Pixel size (Å)			1.09	
Symmetry imposed			C1	
Initial particle images (no.)			723 908	
Final particle images (no.)	294 142	122 228	103 572	
Map resolution (Å)	3.4	3.6	3.7	
FSC threshold	0.143	0.143	0.143	
Map sharpening *B* factor (Å^2)	EM-GAN	DeepEMhancer	DeepEMhancer	DeepEMhancer
**Refinement**				
Initial model used (PDB code)				7OO3, 6PAI, 3EI3, AlphaFold2
Model resolution (Å)				3.4
FSC threshold				0.5
Model composition				
Non-hydrogen atoms				13309
Protein residues				1687
Ligands				0
B factors (Å^2)				
Protein				77.68
Ligand				
R.m.s. deviations				
Bond lengths (Å)				0.006
Bond angles (°)				1.043
Validation				
MolProbity score				1.89
Clashscore				10.30
Poor rotamers (%)				0.34
Ramachandran plot				
Favored (%)				94.91
Allowed (%)				5.09
Disallowed (%)				0.00

Supplementary Table1. Cryo-EM data collection, refinement and validation statistics
