# Supplementary material for: The small CRL4CSA ubiquitin ligase component DDA1 regulates transcription-coupled repair dynamics
Source: Nat Commun. 2024 Jul 29;15:6374. doi: 10.1038/s41467-024-50584-7 (PMC11286758; doi:10.1038/s41467-024-50584-7)
Supplement: Supplementary file 3 — Description of Additional Supplementary Files [file 41467_2024_50584_MOESM3_ESM.pdf]

Description of Additional Supplementary Files

File Name: Supplementary Data 1. CSA-mClover
Description: Table with SILAC ratios as determined using quantitative interaction proteomics (CSA-mClover).

File Name: Supplementary Data 2. GFP-DDB2.
Description: Table with SILAC ratios as determined using quantitative interaction proteomics (GFP-DDB2).

File Name: Supplementary Data 3. XL-MS
Description: Table Crosslinked peptides (XL-MS).

File Name: Supplementary Data 4. RNA-seq CSAKO
Description: Table sequencing data, differential expression in CSAKO versus WT HCT116 cells (RNA-seq). Differentially expression was analyzed using the glmQLFTest. P-values were corrected using Benjamini-Hochberg procedure.

File Name: Supplementary Data 5. RNA-seq DDA1KO
Description: Table sequencing data, differential expression in DDA1KO versus WT HCT116 cells (RNA-seq). Differentially expression was analyzed using the glmQLFTest. P-values were corrected using Benjamini-Hochberg procedure.

File Name: Supplementary Data 6. Total proteins profile
Description: Table with SILAC ratios as determined using quantitative proteomics of total proteins (WT, CSAKO, DDA1KO HCT116 cell lines).

File Name: Supplementary Data 7. DIA CSAmClover
Description: Table label free (Data Independent Analysis) as determined using quantitative interaction proteomics (CSA-mClover in WT and DDA1KO cell lines). Two-sample test (two-tailed) is applied for determining if the means of the LFQ intensity values of samples are significantly different from each other.

File Name: Supplementary Data 8. Ubiquitin profile
Description: Table with SILAC ratios as determined using quantitative proteomics of ubiquitin peptides (WT, CSAKO, DDA1KO HCT116 cell lines).

File Name: Supplementary Data 9. Ubiquitin profile comparison CSAKO/DDA1KO
Description: Table with SILAC ratios as determined using quantitative proteomics of ubiquitin peptides (comparison CSAKO versus DDA1KO HCT116 cell lines).

File Name: Supplementary Data 10. Table Map-road MS data
Description: Table Map-road MS data.

File Name: Supplementary Data 11. Table reagent or resource
Description: Table reagent or resource
