# Supplementary material for: The small CRL4CSA ubiquitin ligase component DDA1 regulates transcription-coupled repair dynamics
Source: Nat Commun. 2024 Jul 29;15:6374. doi: 10.1038/s41467-024-50584-7 (PMC11286758; doi:10.1038/s41467-024-50584-7)
Supplement: Supplementary file 15 — Reporting Summary [file 41467_2024_50584_MOESM15_ESM.pdf]

nature portfolio

Corresponding author(s): Wim Vermeulen and Alex Pines

Last updated by author(s): Jun 18, 2024

Reporting Summary

Nature Portfolio wishes to improve the reproducibility of the work that we publish. This form provides structure for consistency and transparency in reporting. For further information on Nature Portfolio policies, see our Editorial Policies and the Editorial Policy Checklist.

Statistics

For all statistical analyses, confirm that the following items are present in the figure legend, table legend, main text, or Methods section.

n/a	Confirmed	
[ ]	[x]	The exact sample size (*n*) for each experimental group/condition, given as a discrete number and unit of measurement
[ ]	[x]	A statement on whether measurements were taken from distinct samples or whether the same sample was measured repeatedly
[ ]	[x]	The statistical test(s) used AND whether they are one- or two-sided<br>*Only common tests should be described solely by name; describe more complex techniques in the Methods section.*
[x]	[ ]	A description of all covariates tested
[ ]	[x]	A description of any assumptions or corrections, such as tests of normality and adjustment for multiple comparisons
[ ]	[x]	A full description of the statistical parameters including central tendency (e.g. means) or other basic estimates (e.g. regression coefficient) AND variation (e.g. standard deviation) or associated estimates of uncertainty (e.g. confidence intervals)
[ ]	[x]	For null hypothesis testing, the test statistic (e.g. *F*, *t*, *r*) with confidence intervals, effect sizes, degrees of freedom and *P* value noted<br>*Give P values as exact values whenever suitable.*
[x]	[ ]	For Bayesian analysis, information on the choice of priors and Markov chain Monte Carlo settings
[x]	[ ]	For hierarchical and complex designs, identification of the appropriate level for tests and full reporting of outcomes
[x]	[ ]	Estimates of effect sizes (e.g. Cohen's *d*, Pearson's *r*), indicating how they were calculated

Our web collection on statistics for biologists contains articles on many of the points above.

Software and code

Policy information about availability of computer code

Data collection	-Microscopy data was obtained using commercially available Leica LAS AF software or Carl Zeiss LSM software.<br>-Mass spectrometry analyses were performed on a Thermo Scientific Orbitrap Fusion™ Lumos Tribrid™ Mass spectrometer or an Orbitrap Eclipse™ Tribrid™ mass spectrometer directly coupled to the EASYnLC. All mass spectra were acquired in profile mode.<br>-Cryo-EM data were obtained using FEI Titan Krios 300 kV electron microscope with a K3 detector (Gatan) and an energy filter (Gatan) with slit width of 20 eV. Automated data collection was using EPU (ThermoFisher Scientific).<br>-Sequencing was performed on NextSeq 2000, using a 51/8/8 cycle setup to sequence the read 1, first index, and second index respectively.<br>-Differential scanning fluorometry data were obtained using the Prometheus NT.48.<br>-Immunoblotting, the Proteins were visualized by the Odyssey® Imaging System.
Data analysis	-Data was analyzed by Leica LAS AF (version 3.3.0.16799) software, Carl Zeiss LSM (version 14.0.0.0), ImageJ/ Fiji software and further processed in Excel (2016).<br>-Maxquant (version 1.6.3.3 or 2.0.3.0) Spectronaut Pulsar X (version 17.0.221202) were used to analyze quantitative proteomics data. Data was plotted and analyzed using Perseus (version 1.6.14.0) and GraphPad Prism 8.2.1.<br>-Cryo-EM, the initial data processing was carried out in cryoSPARC. Particles were picked using TOPAZ. After 2D classification clean-up and consensus 3D refinement, the coordinates were imported to Relion3.0. In Relion, micrographs were motion corrected by MotionCor2 and the contrast transfer function was estimated by CTFFIND-4.1. Model building and refinement were done in Chimera, Phenix and Coot.<br>-Reads were analyzed using FastQC v0.12.1 and MultiQC v1.18 to determine the overall quality of sequencing. STAR 2.7.11b was used to align reads to genome build hg38 patch 13. QC metrics were extracted using GATK CollectRnaSeqMetrics.

For manuscripts utilizing custom algorithms or software that are central to the research but not yet described in published literature, software must be made available to editors and reviewers. We strongly encourage code deposition in a community repository (e.g. GitHub). See the Nature Portfolio guidelines for submitting code & software for further information.

Data

Policy information about availability of data

All manuscripts must include a data availability statement. This statement should provide the following information, where applicable:

- Accession codes, unique identifiers, or web links for publicly available datasets
- A description of any restrictions on data availability
- For clinical datasets or third party data, please ensure that the statement adheres to our policy

-The SILAC and LQF based quantitative interaction proteomics data have been deposited to the ProteomeXchange Consortium via the PRIDE partner repository with the dataset identifier PXD045415 and PXD051638.
-Cryo-EM maps and atomic models have been deposited in the Electron Microscopy Database and Protein Data Bank respectively under accession codes EMD-18377, EMD-18378, EMD-18380, EMD-18413 and PDB-8QH5.
-The RNA sequencing data have been deposited to the SRA database repository with the dataset identifier PRJNA1103704.

Research involving human participants, their data, or biological material

Policy information about studies with human participants or human data. See also policy information about sex, gender (identity/presentation), and sexual orientation and race, ethnicity and racism.

Reporting on sex and gender	N/A
Reporting on race, ethnicity, or other socially relevant groupings	N/A
Population characteristics	N/A
Recruitment	N/A
Ethics oversight	N/A

Note that full information on the approval of the study protocol must also be provided in the manuscript.

Field-specific reporting

Please select the one below that is the best fit for your research. If you are not sure, read the appropriate sections before making your selection.

☒ Life sciences ☐ Behavioural & social sciences ☐ Ecological, evolutionary & environmental sciences

For a reference copy of the document with all sections, see nature.com/documents/nr-reporting-summary-flat.pdf

Life sciences study design

All studies must disclose on these points even when the disclosure is negative.

Sample size	Sample sizes are specified in the legend to each figure and were chosen based on prior extensive experience in the applied techniques (Geijer 2021, PMID: 34108662; Ribeiro-Silva 2018, PMID: 30287812; Ribeiro-Silva 2020 PMID: 32985517; Wienholz 2019 PMID:30715484; Steurer 2018 PMID: 29632207; van Cuijck 2015, PMID: 26151477; Dinant 2013; PMID: 23973375;), which allows us to estimate beforehand how many samples should be analyzed to provide sufficient statistical power to distinguish real differences.
Data exclusions	No samples were excluded
Replication	Experiments were replicated as indicated per experiment in the legends. All replication attempts were successful.
Randomization	Randomization was not applicable to our study. The assays used in this study are not subject to systematic variation that demands randomization
Blinding	Blinding was not applicable to our study. Blinding in IP / western blot approaches is not possible.

Reporting for specific materials, systems and methods

We require information from authors about some types of materials, experimental systems and methods used in many studies. Here, indicate whether each material, system or method listed is relevant to your study. If you are not sure if a list item applies to your research, read the appropriate section before selecting a response.

Materials & experimental systems

n/a	Involved in the study
☐	☒ Antibodies
☐	☒ Eukaryotic cell lines
☒	☐ Palaeontology and archaeology
☒	☐ Animals and other organisms
☒	☐ Clinical data
☒	☐ Dual use research of concern
☒	☐ Plants

Methods

n/a	Involved in the study
☒	☐ ChIP-seq
☒	☐ Flow cytometry
☒	☐ MRI-based neuroimaging

Antibodies

Antibodies used

Alexa Fluor ® 647 Phalloidin Life Technologies Europe BV A22287 (1:2000 Immunofluorescence)
Alexa Fluor 488 (rabbit) Invitrogen A11008 (1:1000 Immunofluorescence)
Alexa Fluor 594 (mouse) Invitrogen A11005 (1:1000 Immunofluorescence)
Alexa Fluor 594 (rabbit) Invitrogen A21207 (1:1000 Immunofluorescence)
Goat anti-mouse CF™ IRDye 680 Sigma-Aldrich sab4600199 (1:10000 Immunoblotting)
Goat anti-rabbit CF™ IRDye 770 Sigma-Aldrich sab4600215 (1:10000 Immunoblotting)
Mouse anti-CSN5 Novus biologicals NB120-495 (1:1000 Immunoblotting)
Mouse anti-TCP-1 Abnova H00006950-M01 (1:1000 Immunoblotting)
Mouse anti-Tubulin (B512) Sigma-Aldrich T5168 (1:5000 Immunoblotting)
Rabbit anti- Rpb1 NTD Cell Signaling Techn 14958 (1:1000 Immunoblotting)
Rabbit anti-CSA Abcam ab240096, ab137033 (1:1000 Immunoblotting, 1:400 Immunofluorescence)
Rabbit anti-CSB antibodies-online.com ABIN2855858 (1:1000 Immunoblotting)
Rabbit anti-CUL4A Abcam ab72548 (1:1000 Immunoblotting)
Rabbit anti-DDA1 antibodies-online.com ABIN2798422 (1:200 Immunoblotting)
Rabbit anti-DDB1 Novus biologicals NBP2-75465 (1:1000 Immunoblotting)
Rabbit anti-GFP Abcam ab290 (1:1000 Immunoblotting)
Rabbit anti-H2B Millipore 07-371 (1:1000 Immunoblotting)

Validation

Antibodies were validated as indicated on their manufacturer's website, where validated in previous publications of our lab by siRNA/KO experiments or where checked by western blot or immunofluorescence in this manuscript, mostly with a siRNA/KO as control for specificity. All the antibodies used in the manuscript showed bands of expected size.
Mouse anti-CSN5 Novus biologicals NB120-495 was previously validated by the manufacturer (https://www.novusbio.com/products/jab1-antibody-2a108_nb120-495)
Mouse anti-TCP-1 Abnova H00006950-M01 verified in our lab PMID: 29531219
Mouse anti-Tubulin (B512) Sigma-Aldrich T5168, commonly used a loading control in the lab, verified by specific and intense band at correct height
Rabbit anti- Rpb1 NTD Cell Signaling Techn 14958 verified in our lab PMID: 37716192
Rabbit anti-CSA Abcam ab240096, ab137033 verified in our lab PMID: 29531219 and PMID: 37716192
Rabbit anti-CSB antibodies-online.com ABIN2855858 verified in our lab PMID: 37716192
Rabbit anti-CUL4A Abcam ab72548 was previously validated by the manufacturer (https://www.abcam.com/en-it/products/primary-antibodies/anti-cullin-4a-cul-4a-antibody-ab72548)
Rabbit anti-DDA1 antibodies-online.com ABIN2798422 verified by KO and siRNA, immunoblotting data fig3A (DDA1KO) and Extended data fig5B (siRNA)
Rabbit anti-DDB1 Novus biologicals NBP2-75465 verified in our lab PMID: 37716192
Rabbit anti-GFP Abcam ab290 was previously validated by the manufacturer (https://www.abcam.com/en-nl/products/primary-antibodies/anti-gfp-antibody-ab290)
Rabbit anti-H2B Millipore 07-371 verified in our lab PMID: 37716192

Eukaryotic cell lines

Policy information about cell lines and Sex and Gender in Research

Cell line source(s)

HCT116 from Horizon Discovery
VH10 fibroblasts (hTert), VH10 fibroblasts (hTert) GFP-DDB2, CS3BE (CS-A, hTert), CS3BE CSA-GFP(CS-A, hTert), HCT116 CSAKO, HCT116 CSB KO and HCT116 CSB-mClover cells are routinely used in the host institute as described in PMID: 29531219, PMID: 23045548 and PMID: 37716192

Authentication

None were authenticated.

Mycoplasma contamination

All cell lines were routinely tested for mycoplasma and were all negative.

Commonly misidentified lines
(See ICLAC register)

No commonly misidentified cell lines were used in the study.
