# Supplementary material for: The small CRL4CSA ubiquitin ligase component DDA1 regulates transcription-coupled repair dynamics
Source: Nat Commun. 2024 Jul 29;15:6374. doi: 10.1038/s41467-024-50584-7 (PMC11286758; doi:10.1038/s41467-024-50584-7)
Supplement: Supplementary file 16 — Source Data [file 41467_2024_50584_MOESM16_ESM.zip › Source data/Supplementary Fig6/Supplentary Fig6 A-B.docx]

Supplementary Fig6:

Deposite CryoEM data: EMDB (**EMD-18377; EMD-18378; EMD-18380; EMD-18413**), PDB (**PDB-8QH5**)
