# Supplementary figures and images for: The small CRL4CSA ubiquitin ligase component DDA1 regulates transcription-coupled repair dynamics
Source: Nat Commun. 2024 Jul 29;15:6374. doi: 10.1038/s41467-024-50584-7 (PMC11286758; doi:10.1038/s41467-024-50584-7)

Figure 1C

GEL1

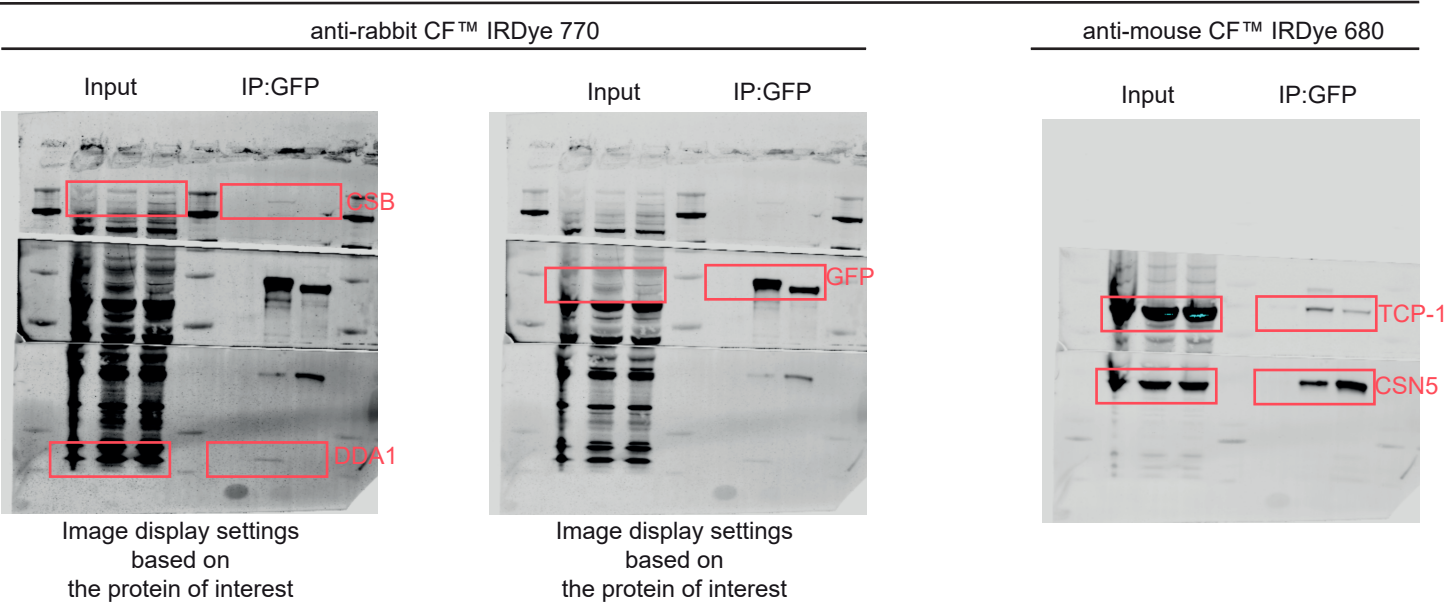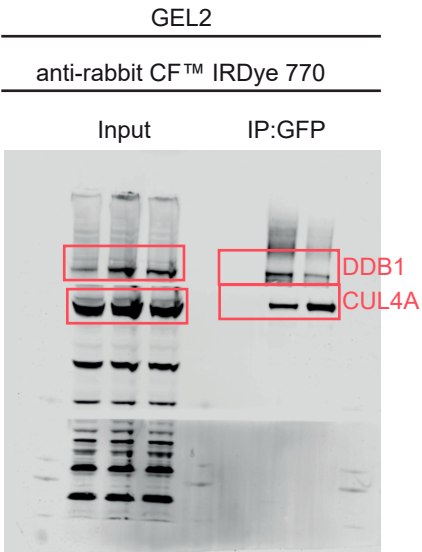

Supplement: Supplementary file 16 — Source Data [file 41467_2024_50584_MOESM16_ESM.zip › Source data/Fig1/Fig1 C.pdf]

Figure 3A

anti-rabbit CF™ IRDye 770

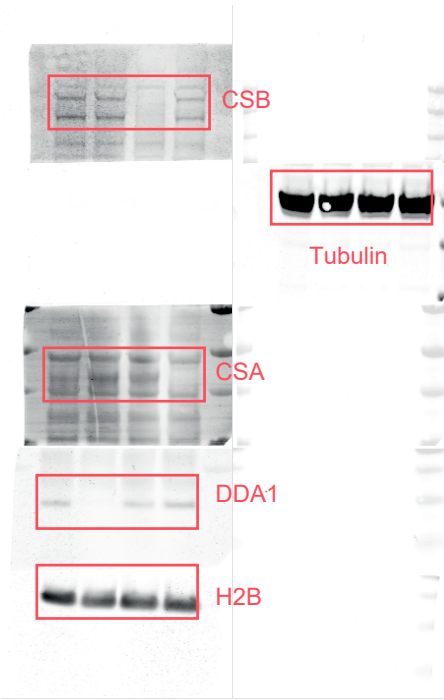

Supplement: Supplementary file 16 — Source Data [file 41467_2024_50584_MOESM16_ESM.zip › Source data/Fig3/Fig3A.pdf]

Figure 6E

GEL1

anti-rabbit CF™ IRDye 770

Input

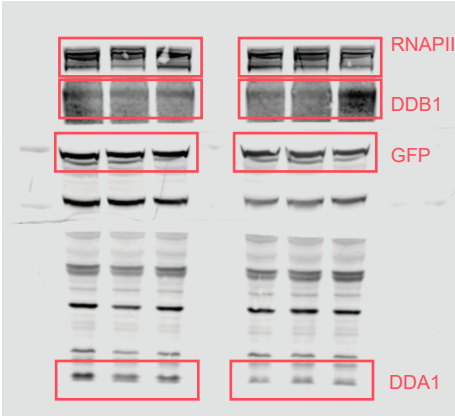

GEL2

anti-rabbit CF™ IRDye 770

IP:GFP

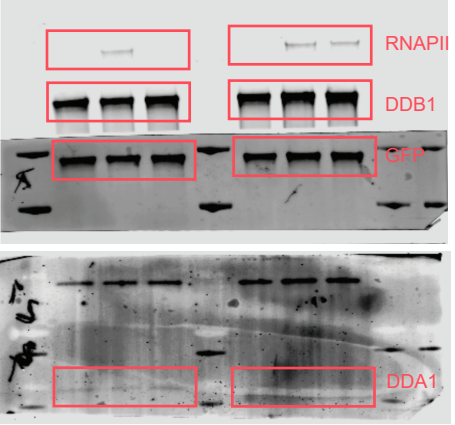

Supplement: Supplementary file 16 — Source Data [file 41467_2024_50584_MOESM16_ESM.zip › Source data/Fig6/Fig6E.pdf]

anti-rabbit CF™ IRDye 770

anti-mouse CF™ IRDye 680

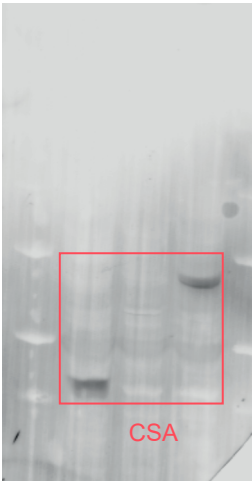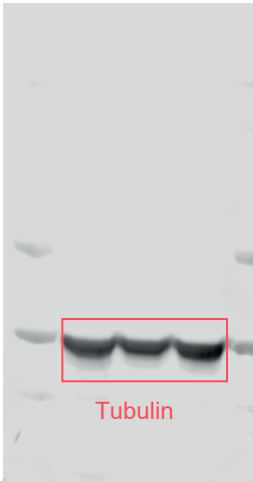

Supplement: Supplementary file 16 — Source Data [file 41467_2024_50584_MOESM16_ESM.zip › Source data/Supplementary Fig1/Supplentary Fig1 B.pdf]

IP:1 No Damage

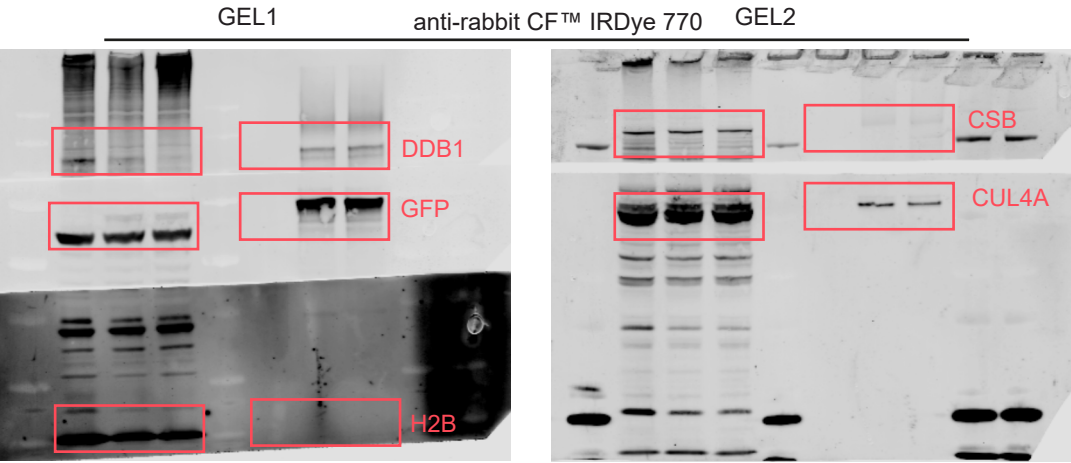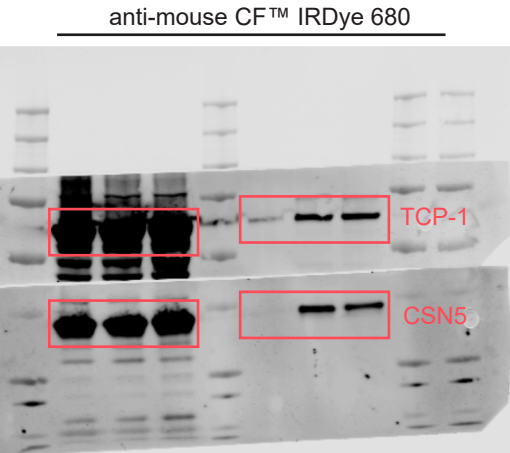

IP:1 +UV

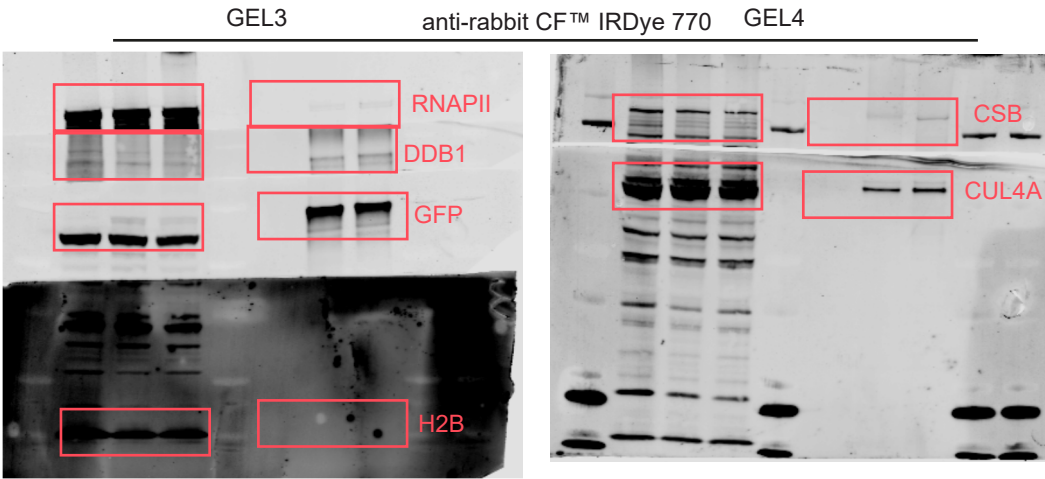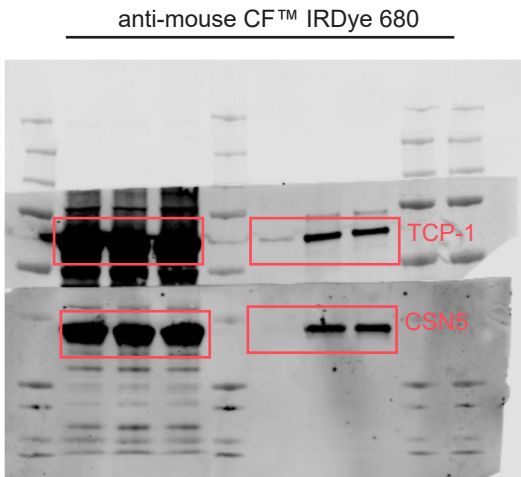

Supplement: Supplementary file 16 — Source Data [file 41467_2024_50584_MOESM16_ESM.zip › Source data/Supplementary Fig11/Raw IP1.pdf]

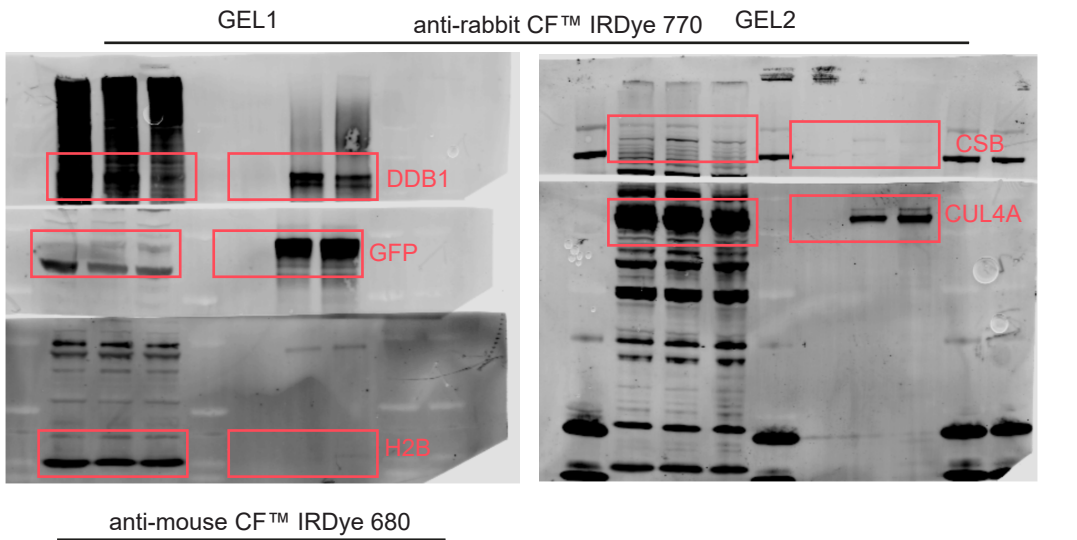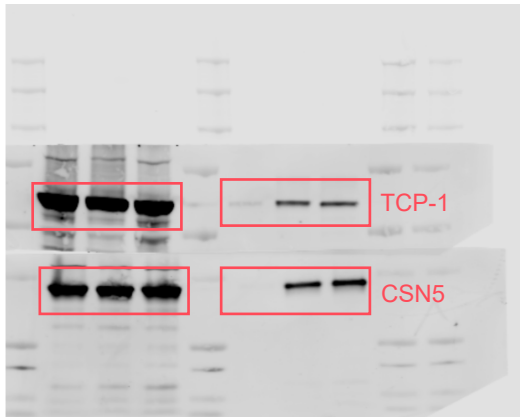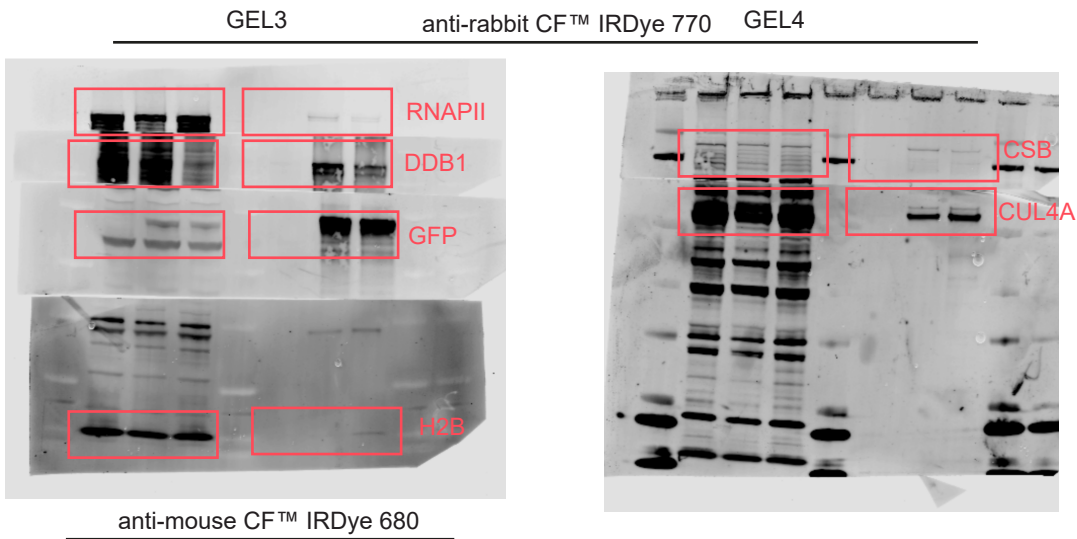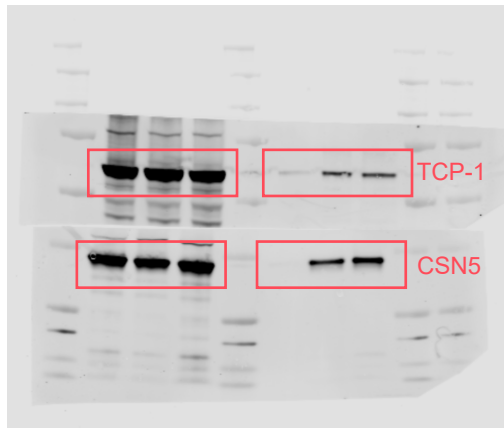

Supplement: Supplementary file 16 — Source Data [file 41467_2024_50584_MOESM16_ESM.zip › Source data/Supplementary Fig11/Raw IP2.pdf]

GEL1 anti-rabbit CF™ IRDye 770 GEL2

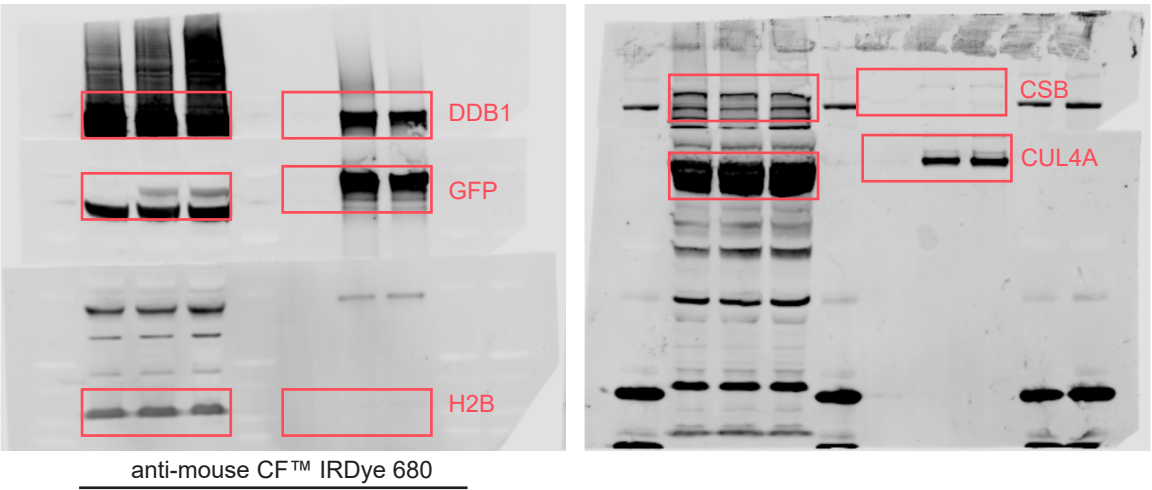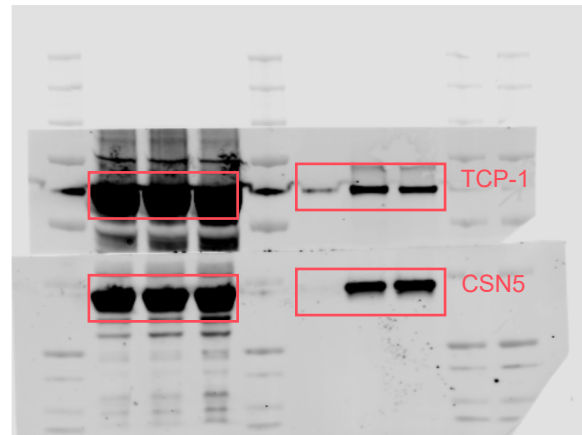

IP:3 +UV

GEL3 anti-rabbit CF™ IRDye 770 GEL4

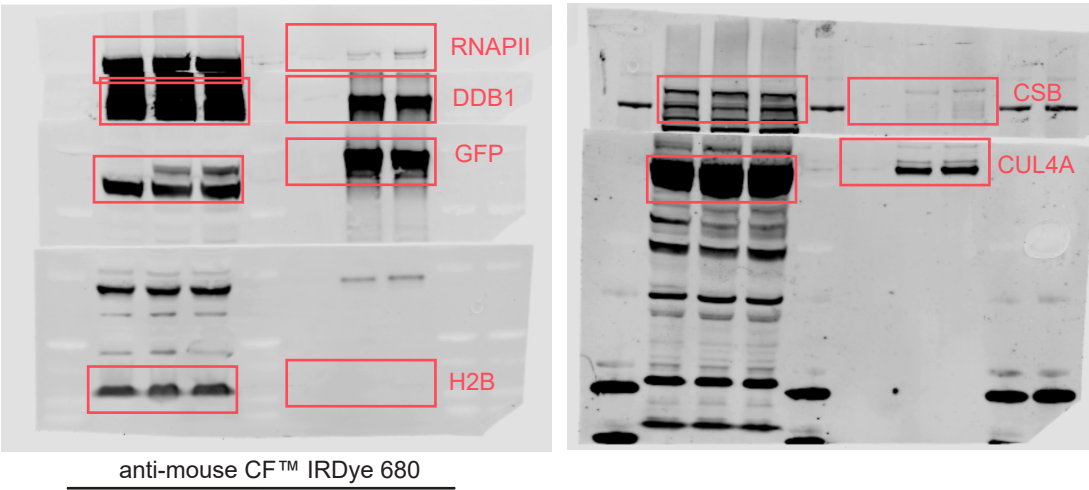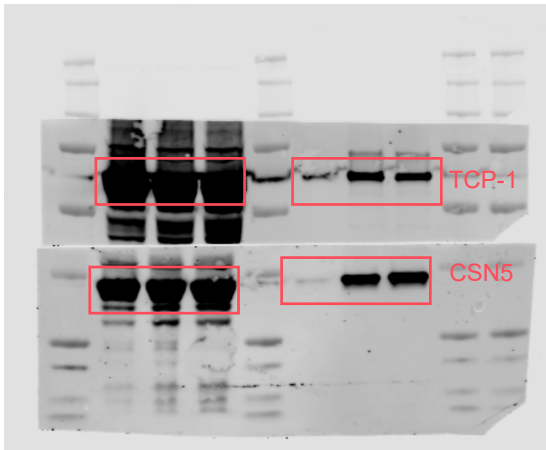

Supplement: Supplementary file 16 — Source Data [file 41467_2024_50584_MOESM16_ESM.zip › Source data/Supplementary Fig11/Raw IP3.pdf]

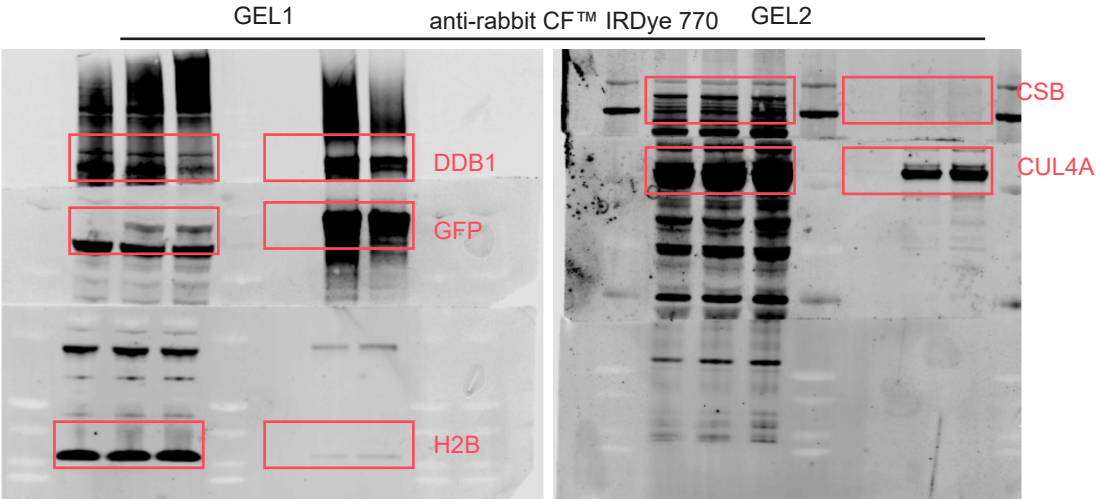

anti-mouse CF™ IRDye 680

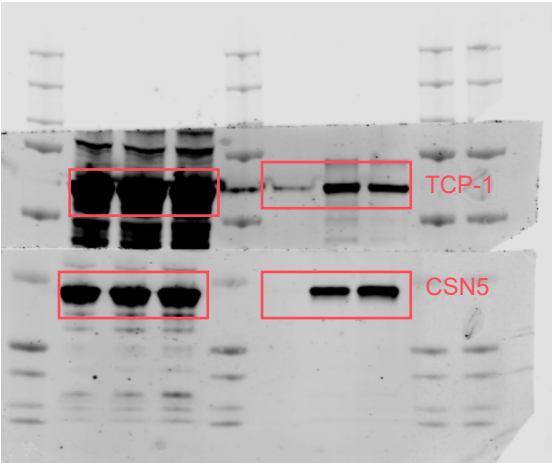

IP:4 +UV

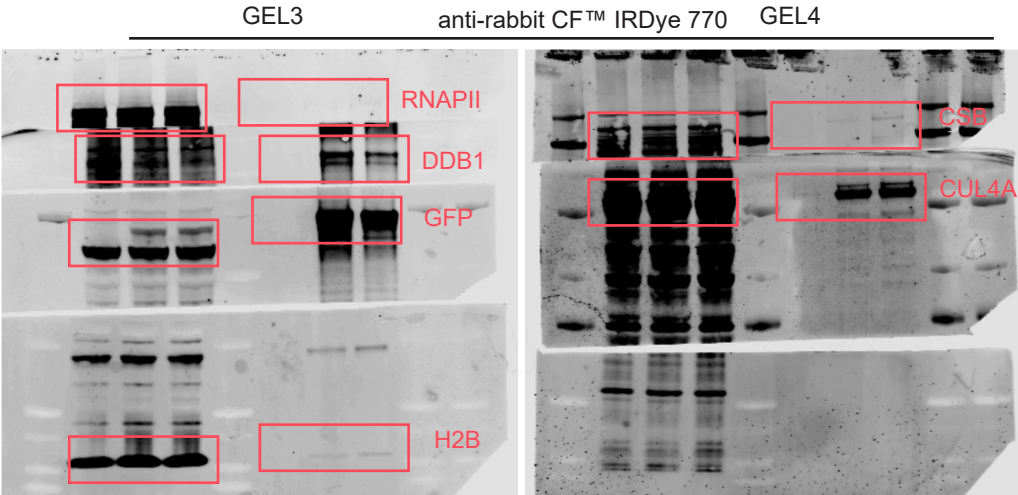

anti-mouse CF™ IRDye 680

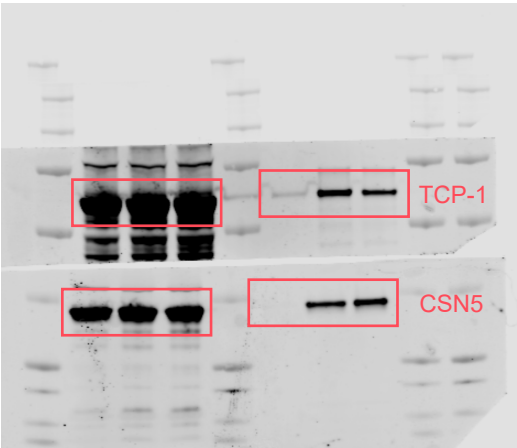

Supplement: Supplementary file 16 — Source Data [file 41467_2024_50584_MOESM16_ESM.zip › Source data/Supplementary Fig11/Raw IP4.pdf]

GEL1 anti-rabbit CF™ IRDye 770 GEL2

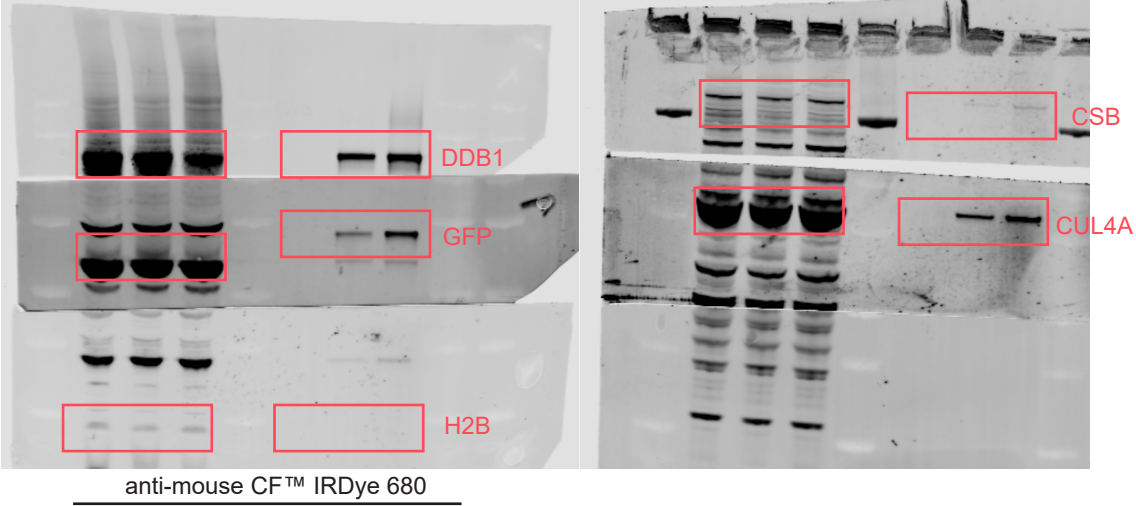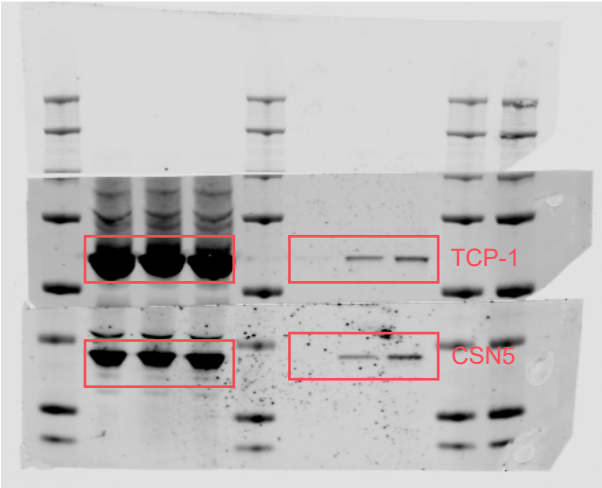

IP:5 +UV

GEL3 anti-rabbit CF™ IRDye 770 GEL4

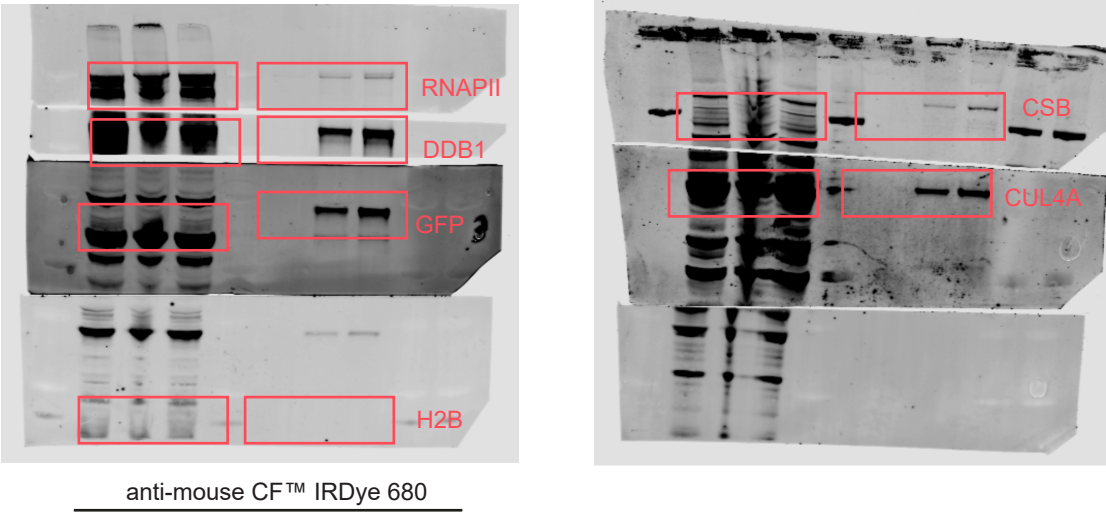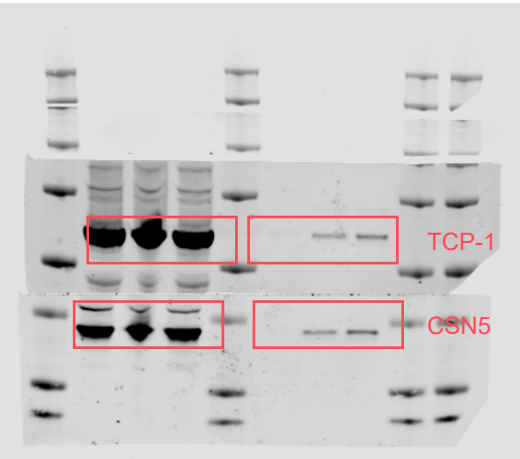

Supplement: Supplementary file 16 — Source Data [file 41467_2024_50584_MOESM16_ESM.zip › Source data/Supplementary Fig11/Raw IP5.pdf]

Supplementary Fig 15 B

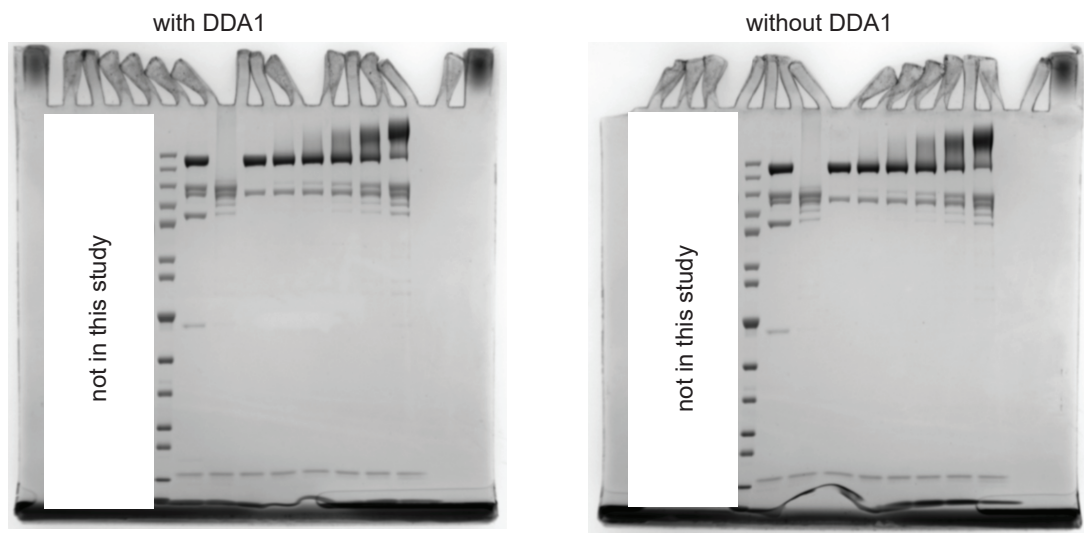

Supplementary Fig 15 C

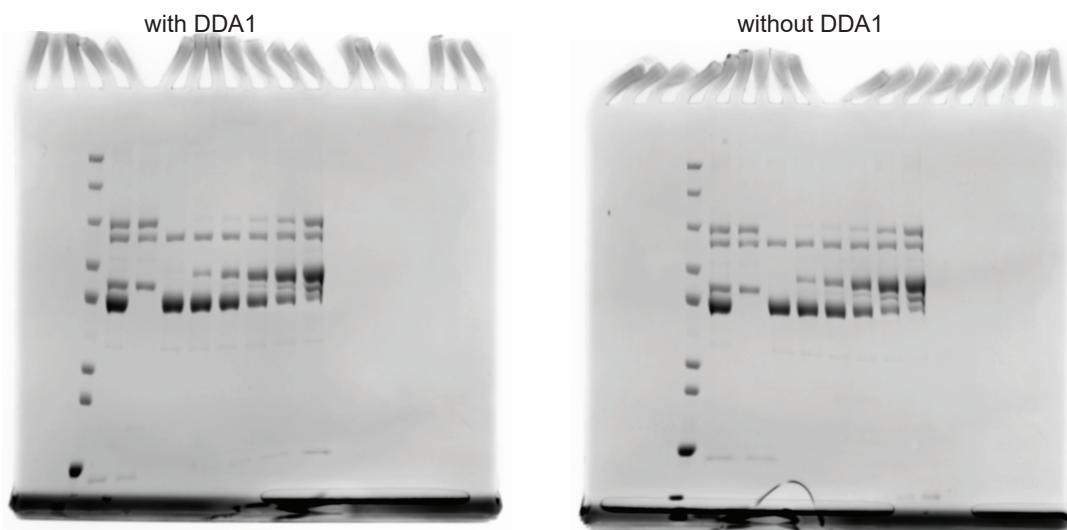

Supplement: Supplementary file 16 — Source Data [file 41467_2024_50584_MOESM16_ESM.zip › Source data/Supplementary Fig15/Supplentary Fig15 A-C.pdf]

Supplementary Figure 2A

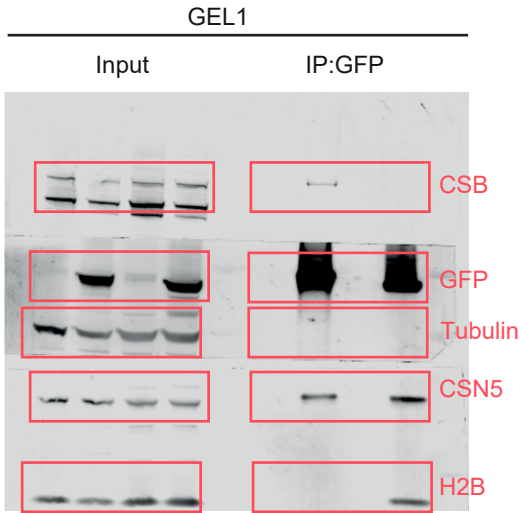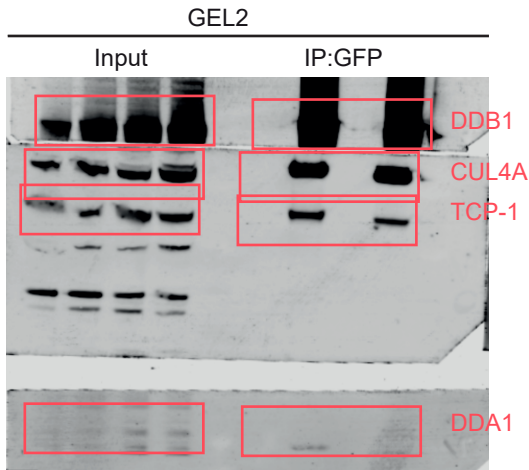

Supplement: Supplementary file 16 — Source Data [file 41467_2024_50584_MOESM16_ESM.zip › Source data/Supplementary Fig2/Supplentary Fig2A.pdf]

anti-rabbit CF™ IRDye 770

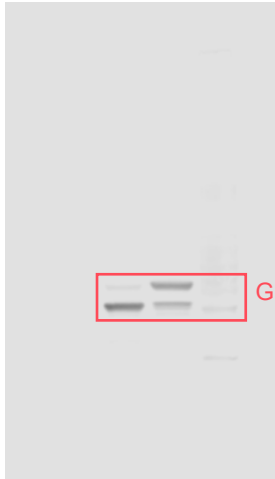

GFP

anti-mouse CF™ IRDye 680

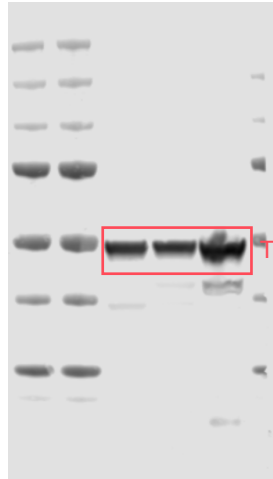

Tubulin

Supplement: Supplementary file 16 — Source Data [file 41467_2024_50584_MOESM16_ESM.zip › Source data/Supplementary Fig7/Supplentary Fig7 D.pdf]

anti-rabbit CF™ IRDye 770

anti-mouse CF™ IRDye 680

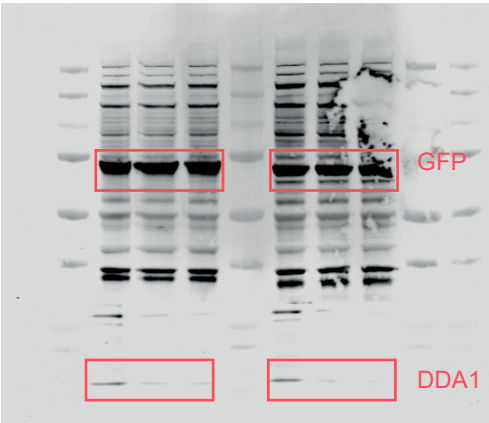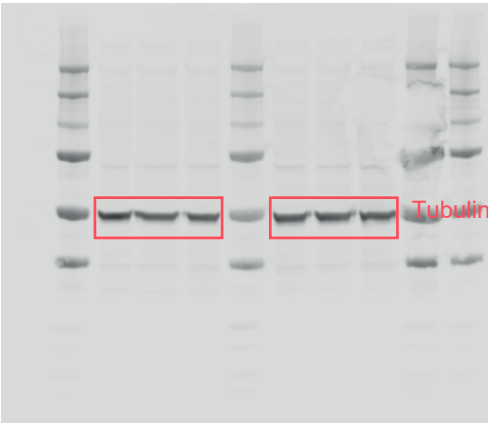

Supplement: Supplementary file 16 — Source Data [file 41467_2024_50584_MOESM16_ESM.zip › Source data/Supplementary Fig9/Supplentary Fig9 A.pdf]
