# Supplementary material for: The small CRL4CSA ubiquitin ligase component DDA1 regulates transcription-coupled repair dynamics
Source: Nat Commun. 2024 Jul 29;15:6374. doi: 10.1038/s41467-024-50584-7 (PMC11286758; doi:10.1038/s41467-024-50584-7)
Supplement: Supplementary file 16 — Source Data [file 41467_2024_50584_MOESM16_ESM.zip › Source data/Fig2/cryoEM_table.doc]

**Cryo-EM data collection, refinement and validation statistics**

|  | CSA-DDB1-DDA1  (Map 1)  (EMDB-18377) | CSA-DDB1-DDA1  (Map 2)  (EMDB-18378) | CSA-DDB1^BPA/BPC^-DDA1-UVSSA^VHS^  (Map 3)  (EMDB-18380) | | | CSA-DDB1-DDA1-UVSSA^VHS^  (composite map)  (EMDB-18398)  (PDB-8QH5) | | |  | | | |
| --- | --- | --- | --- | --- | --- | --- | --- | --- | --- | --- | --- | --- |
| **Data collection and processing** |  |  | |  | | |  | | |  | | |
| Magnification | 81 000× | | | | | | | | | | | |
| Voltage (kV) | 300 | | | | | | | | | | | |
| Electron exposure (e–/Å^2^) | 60 | | | | | | | | | | | |
| Defocus range (μm) | -1.0 to -3.0 | | | | | | | | | | | |
| Pixel size (Å) | 1.09 | | | | | | | | | | | |
| Symmetry imposed | C1 | | | | | | | | | | | |
| Initial particle images (no.) | 723 908 | | | | | | | | | | | |
| Final particle images (no.) | 294 142 | 122 228 | | 103 572 | | |  | | |  | | |
| Map resolution (Å)  FSC threshold | 3.4  0.143 | 3.6  0.143 | | 3.7  0.143 | | |  | | |  | | |
| Map sharpening *B* factor (Å^2^) | EM-GAN | DeepEMhancer | | DeepEMhancer | | | DeepEMhancer | | |  | | |
|  |  | | | | | | | | | | |  |
| **Refinement** |  |  | | |  | | |  | | |  | |
| Initial model used  (PDB code) |  |  | | |  | | | 7OO3, 6PAI, 3EI3, AlphaFold2 | | |  | |
| Model resolution (Å)  FSC threshold |  |  | | |  | | | 3.4  0.5 | | |  | |
| Model composition  Non-hydrogen atoms  Protein residues  Ligands |  |  | | |  | | | 13309  1687  0 | | |  | |
| *B* factors (Å^2^)  Protein  Ligand |  |  | | |  | | | 77.68 | | |  | |
| R.m.s. deviations  Bond lengths (Å)  Bond angles (°) |  |  | | |  | | | 0.006  1.043 | | |  | |
| Validation  MolProbity score  Clashscore  Poor rotamers (%) |  |  | | |  | | | 1.89  10.30  0.34 | | |  | |
| Ramachandran plot  Favored (%)  Allowed (%)  Disallowed (%) |  |  | | |  | | | 94.91  5.09  0.00 | | |  | |
|  |  |  | | |  | | |  | | |  | |
|  |  |  | | |  | | |  | | |  | |
|  |  |  | | |  | | |  | | |  | |
